# A Review: Application of Doped Hydrogenated Nanocrystalline Silicon Oxide in High Efficiency Solar Cell Devices

**DOI:** 10.1002/advs.202403728

**Published:** 2024-07-18

**Authors:** Depeng Qiu, Andreas Lambertz, Weiyuan Duan, Luana Mazzarella, Philipp Wagner, Anna Belen Morales‐Vilches, Guangtao Yang, Paul Procel, Olindo Isabella, Bernd Stannowski, Kaining Ding

**Affiliations:** ^1^ Institute of Energy Research Jiangxi Academy of Sciences Nanchang 330096 China; ^2^ IEK‐5 Photovoltaics Forschungszentrum Jülich GmbH, Wilhelm‐Johnen Straße 52425 Jülich Germany; ^3^ Photovoltaic Materials and Devices Group Delft University of Technology Mekelweg 4 Delft 2628 CD The Netherlands; ^4^ Solar Energy Division, Department Perovskite Tandem Solar Cells Helmholtz‐Zentrum Berlin 12489 Berlin Germany; ^5^ Solar Energy Division Competence Centre Photovoltaics Berlin (PVcomB) Helmholtz‐Zentrum Berlin 12489 Berlin Germany; ^6^ Trina Solar Co., Ltd. No. 2, TianHe Road, TrinaPV Industrial Park, Xinbei District Changzhou Jiangsu 213000 China; ^7^ Carbon Neutrality Research Center of Jiangxi Province Nanchang 330096 China; ^8^ Key Laboratory of Greenhouse Gas Accounting and Carbon Reduction of Jiangxi Province Nanchang 330096 China

**Keywords:** applications, carrier transport, hydrogenated nanocrystalline silicon oxide, solar cell

## Abstract

Due to the unique microstructure of hydrogenated nanocrystalline silicon oxide (nc‐SiO_x_:H), the optoelectronic properties of this material can be tuned over a wide range, which makes it adaptable to different solar cell applications. In this work, the authors review the material properties of nc‐SiO_x_:H and the versatility of its applications in different types of solar cells. The review starts by introducing the growth principle of doped nc‐SiO_x_:H layers, the effect of oxygen content on the material properties, and the relationship between optoelectronic properties and its microstructure. A theoretical analysis of charge carrier transport mechanisms in silicon heterojunction (SHJ) solar cells with wide band gap layers is then presented. Afterwards, the authors focus on the recent developments in the implementation of nc‐SiO_x_:H and hydrogenated amorphous silicon oxide (a‐SiO_x_:H) films for SHJ, passivating contacts, and perovskite/silicon tandem devices.

## Graphical abstract

The authors review the material properties of and the versatility of its applications in different types of solar cells. The review starts by introducing the growth principle of doped hydrogenated nanocrystalline silicon oxide (nc‐SiO_x_:H) layers and then presents a theoretical analysis of charge carrier transport mechanisms in silicon heterojunction (SHJ) solar cells with wide band gap layers. Furthermore, the authors focus on the recent developments in the implementation of nc‐SiO_x_:H nd amorphous silicon oxide films for SHJ, passivating contacts, and perovskite/silicon tandem devices.

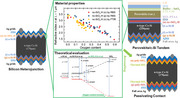

## 1 Introduction

In recent years, doped hydrogenated nanocrystalline silicon oxide (nc‐SiO_x_:H) has been investigated for its application in high efficiency crystalline silicon (c‐Si) solar cells^[^ ^1^, ^2^, ^3^, ^4^, ^5^, ^6^, ^7^, ^8^ ^]^ and perovskite/c‐Si tandem solar cells.^[^ ^9^, ^10^, ^11^, ^12^, ^13^, ^14^, ^15^, ^16^ ^]^ As a mixed phase material, the optical and electrical properties can be tuned over a wide range, making it adaptable to different solar cell applications.^[^ ^17^, ^18^, ^19^ ^]^ With an optical band gap (*E*_04_) of up to 2.95 eV, nc‐SiO_x_:H features a low optical parasitic absorption when used as a window layer on the front side.^[^ ^16^, ^17^, ^18^, ^19^, ^20^, ^21^, ^22^ ^]^ The wide range of refractive index at 1 µm from 1.5 to over 3.5 allows fine tuning as an interlayer for tandem solar cells.^[^ ^16^, ^17^, ^18^, ^19^, ^20^, ^21^, ^22^ ^]^ In addition, nc‐SiO_x_:H thin films act as good contact materials for solar cell applications due to its dark conductivity (*σ*) of up to 10 (Ω cm)^−1[^ ^23^, ^24^, ^25^, ^26^, ^27^, ^28^ ^]^ and contact resistivity as low as 34.6 mΩ cm^2^ for n‐type contact^[^ ^7^ ^]^ or 5 mΩ cm^2^ for p‐type contact.^[^ ^8^ ^]^

**Figure 1:**
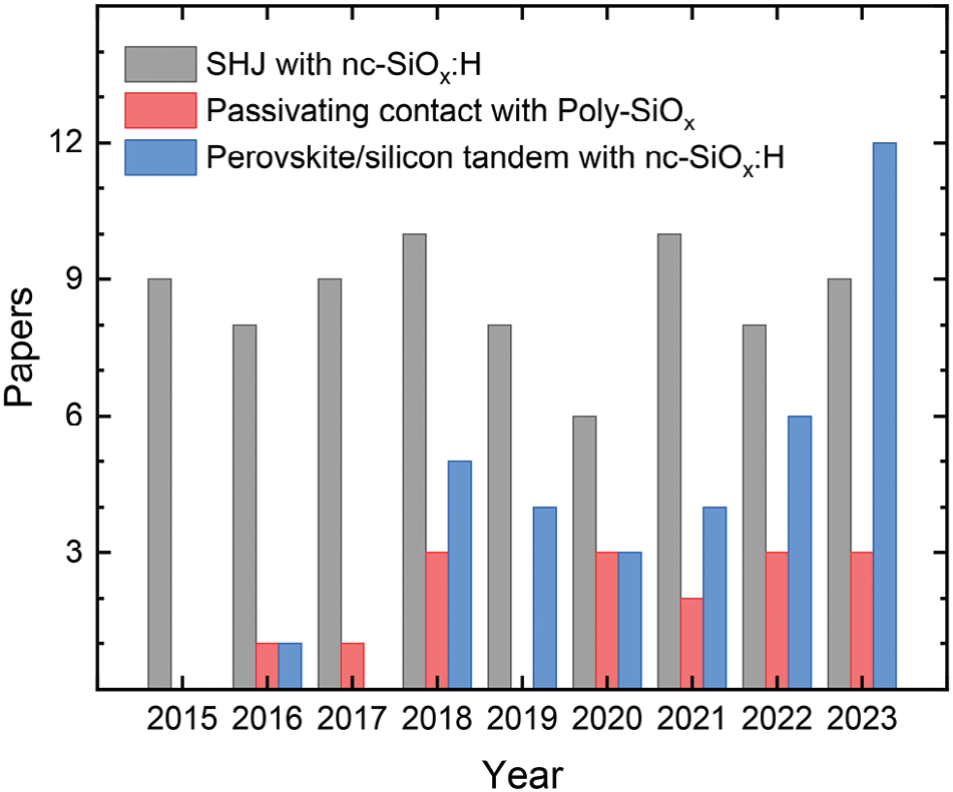
Number of publications on nc‐SiO_x_:H thin films application to SHJ, passivating contact, and perovskite/c‐Si tandem solar cell versus the year of publication. This result is based on research in google scholar with key words such as SHJ, microcrystalline silicon oxide, nanocrystalline silicon oxide, nanocrystalline silicon, poly‐silicon oxide, passivating contact, and perovskite tandem.

Due to these properties of nc‐SiO_x_:H, it has been used on silicon thin‐film solar cells and silicon heterojunction (SHJ) solar cells for years.^[^ ^28^, ^29^, ^30^, ^31^ ^]^ Besides, nc‐SiO_x_:H could also be a good choice for light management in ultra‐thin silicon solar cells, which are attractive for their flexibility and high market potential in PV industry.^[^ ^32^, ^33^, ^34^ ^]^ In recent years new applications such as perovskite/c‐Si tandem solar cells, have been reported.^[^ ^13^, ^14^, ^35^, ^36^, ^37^, ^38^, ^39^, ^40^ ^]^ Record high efficiencies of 26.8%, 27.3%, and 34.6% have been achieved on two‐side contacted SHJ, one‐side contacted SHJ and perovskite/c‐Si tandem solar cells, respectively.^[^ ^41^, ^42^, ^43^ ^]^ In addition, nc‐SiO_x_:H is also deposited upon the tunneling silicon oxide as the precursors for passivating contact devices with polycrystalline silicon oxide (poly‐SiO_x_) films, giving rise to an excellent passivation performance indicated by an implied open‐circuit voltage (i*V*_oc_) of 740 mV.^[^ ^44^ ^]^ It is notable that poly‐SiO_x_ is different from semi‐insulated polycrystalline silicon (SiPOS) films.^[^ ^45^ ^]^ A summary of the number of publications of nc‐SiO_x_:H thin film on these applications based on research in google scholar with key words such as SHJ, microcrystalline silicon oxide, nanocrystalline silicon oxide, nanocrystalline silicon, polysilicon oxide, passivating contact, and perovskite tandem, is shown in **Figure** **1**. It mirrors that nc‐SiO_x_:H is becoming increasingly important for perovskite/silicon tandem applications.

This work reviews the development of doped nc‐SiO_x_:H and its applications on solar cell devices. The growth mechanism is discussed, and the optoelectronic material properties are introduced. We report on the recent progresses of nc‐SiO_x_:H thin films on solar cell applications including SHJ solar cells, tunneling oxide passivating contacts, and perovskite/silicon tandem solar cells. Theory analysis on the device performance applying nc‐SiO_x_:H is discussed by simulation, as well.

## 2 Material Properties

**Figure 2:**
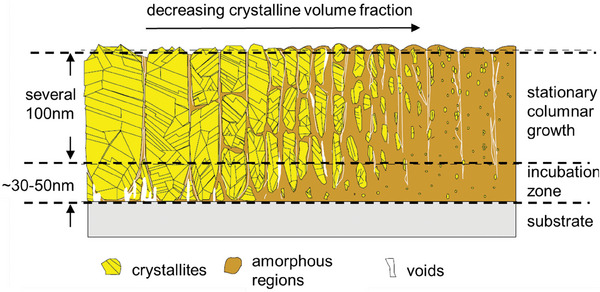
Schematic sketch of the nc‐SiO_x_:H growth. Figure adapted from ref. [50]

The nc‐SiO_x_:H films were deposited via the plasma enhanced chemical vapor deposition (PECVD) for the first time in 1993 by adding CO_2_ during the deposition process of doped nc‐Si:H layer.^[^ ^46^ ^]^ Due to the incorporation of oxygen nc‐SiO_x_:H consists of three phases: nanocrystalline silicon (nc‐Si:H), amorphous silicon (a‐Si:H) and amorphous silicon oxide (a‐SiO_x_:H) phases.^[^ ^17^, ^28^, ^47^, ^48^, ^49^ ^]^ The growth of nc‐SiO_x_:H thin film is depicted in **Figure** **2**.^[^ ^50^ ^]^ An incubation layer and a nucleation layer are grown at the early stage of thin film growth, which is mainly a mixture of a‐Si:H and a‐SiO_x_:H.^[^ ^20^, ^22^, ^51^, ^52^, ^53^ ^]^ The nucleation layer is followed by a growth of cone‐shape crystal of nc‐Si:H until the growth becomes stationary.^[^ ^50^, ^51^, ^52^, ^54^ ^]^ The good conductivity of nc‐SiO_x_:H is attributed to the doped nc‐Si:H phases, while the low refractive index and the high transparency are associated with the a‐SiO_x_:H phases. Hydrogen is preferentially located in the amorphous phase or at the crystal grain boundaries due to the low solubility of H into the crystalline phase^[^ ^55^ ^]^ and is essential for the passivation of the defects.

**Figure 3:**
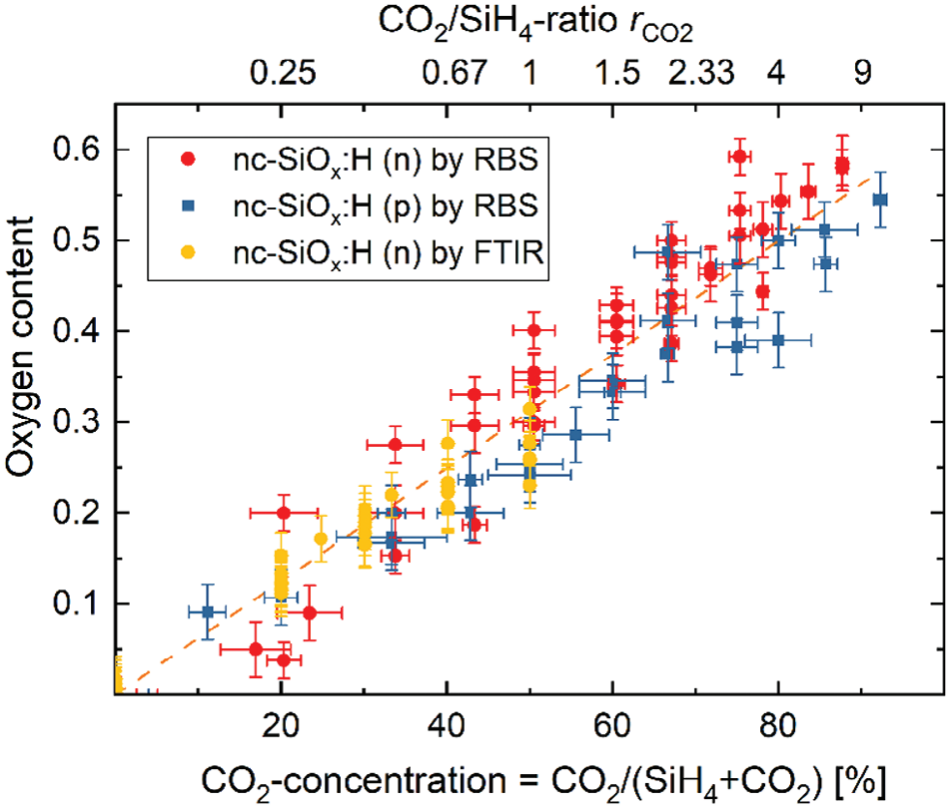
The oxygen content [O] (atomic density) versus the CO_2_ / SiH_4_ mass flow ratio *r*_CO2_ and the corresponding CO_2_‐concentration for n‐type (circles) and p‐type (squares) nc‐SiO_x_:H films.^[^ ^47^, ^57^ ^]^ The values of the O‐content are measured using RBS (red round and blue square symbol) and FTIR (yellow round symbol), for n‐type (phosphorus doped) and p‐type layers (boron doped). The linear regression (dashed line) is fitted to the data of the oxygen content [O] versus the CO_2_ concentration for both types of samples. The CO_2_ concentration in the gas phase during the deposition is defined as CO_2_/(CO_2_ + SiH_4_) mass flows in sccm.

The oxygen content (atomic density) in nc‐SiO_x_:H thin film can be adjusted by the CO_2_ gas flow during the PECVD process.^[^ ^47^, ^56^, ^57^ ^]^ Here, the CO_2_ concentration is the ratio of CO_2_ gas flow rate to the sum of CO_2_ and silane (SiH_4_) flow rate. As shown in **Figure** **3**, increasing the CO_2_ concentration increases the oxygen content of the layer. The oxygen content in silicon was measured using Rutherford backscattering spectroscopy (RBS)^[^ ^47^ ^]^ or Fourier transform infrared (FTIR) spectroscopy,^[^ ^57^ ^]^ and both methods fit well in the trend. Besides the CO_2_ concentration, deposition parameters such as substrate temperature,^[^ ^58^, ^59^ ^]^ pressure,^[^ ^60^ ^]^ hydrogen flow ratio^[^ ^61^ ^]^ are also used to explore the influences on the material properties.

**Figure 4:**
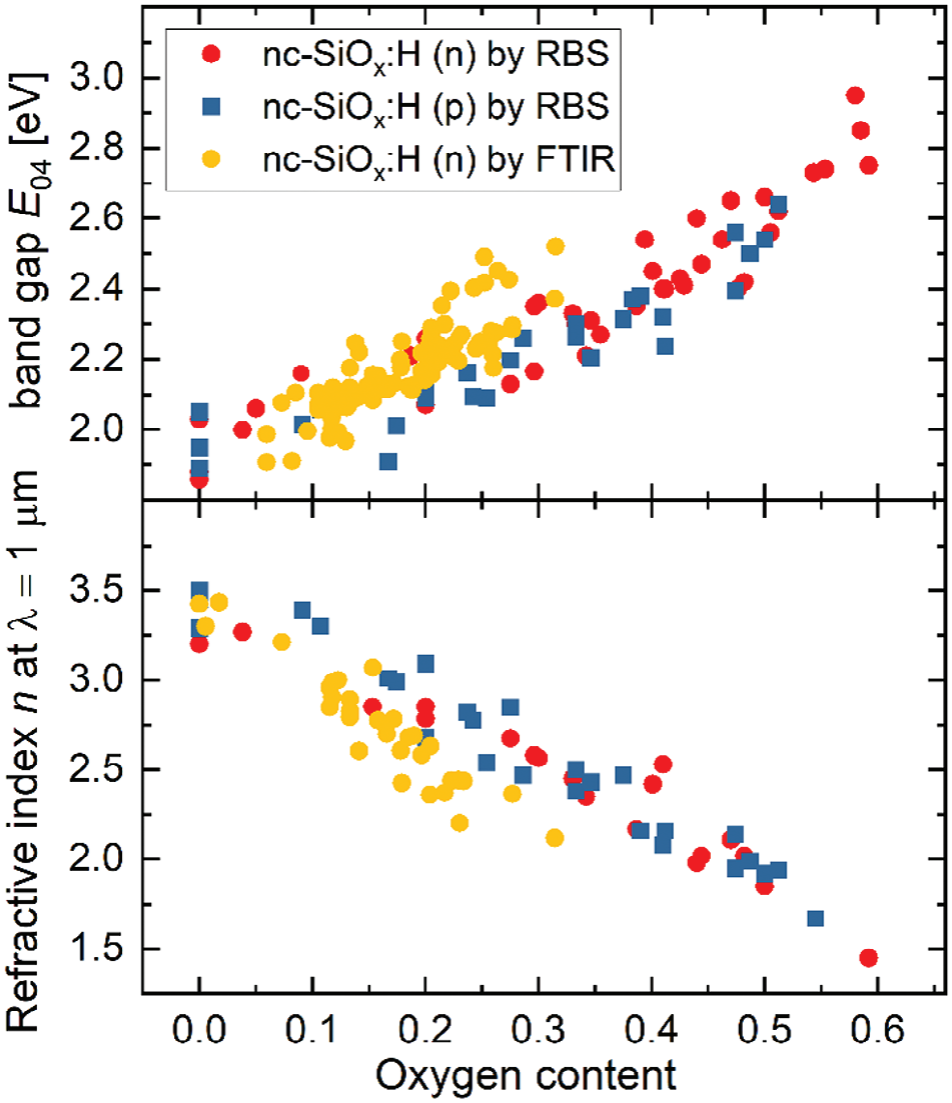
The *E*_04_ band gap and the refractive index plotted versus the oxygen content for n‐type and p‐type nc‐SiO_x_:H films.^[^ ^47^, ^57^ ^]^ The values of the O‐content are measured using RBS (red round and blue square symbol) and FTIR (yellow round symbol), for n‐type (phosphorus doped) and p‐type layers (boron doped).

Being able to control the opto‐electronical properties of nc‐SiO_x_:H is important to achieve high efficiencies for solar cell applications. **Figure** **4** shows the *E*_04_ band gap, and the refractive index plotted versus the oxygen content for n‐type and p‐type nc‐SiO_x_:H films.^[^ ^47^, ^57^ ^]^ Increasing the oxygen content in nc‐SiO_x_:H increases the optical band gap and decreases the refractive index. The oxygen content in the layer was adjusted by varying the CO_2_ concentration during the deposition as shown in Figure 3. The *E*_04_ increases from 1.9 to 2.95 eV and the refractive index decreases from ≈3.5 to 1.5 with increasing oxygen content from 0 to 0.6. From this, one can conclude that the optical properties are largely determined by the oxygen content. It is worth noting that the optical properties show similar dependencies on the oxygen content independent of the doping gas concentration and the silane gas concentration during deposition.^[^ ^22^, ^28^, ^47^, ^53^, ^62^ ^]^

**Figure 5:**
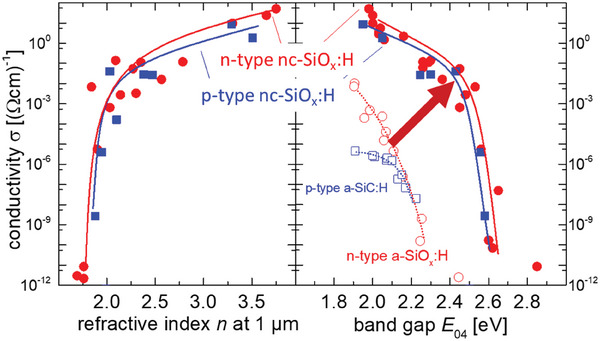
Conductivity versus band gap and conductivity versus refractive index. Electrical and optical properties of several n‐ (circles) and p‐type (squares) nc‐SiO_x_:H films deposited on glass.^[^ ^17^ ^]^ The properties of p‐type a‐SiC:H layers (open squares) and n‐type a‐SiO_x_:H layers (open circles) are shown for comparison.^[^ ^63^ ^]^ The added lines serve as guides to the eye and roughly outline the performance limits of the films.

The conductivity is plotted versus the refractive index and the optical band gap *E*_04_ as shown in **Figure** **5** as a figure of merrit.^[^ ^17^ ^]^ The conductivity is adjustable from 10^−10^ to 10 (Ω cm)^−1^ for both p‐type and n‐type nc‐SiO_x_:H layers. A similar trend was shown for both types of doping that the conductivity increases with increasing refractive index and decreasing band gap. The opto‐electronical properties of nc‐SiO_x_:H are much improved as compared to other doped amorphous silicon alloys.

## 3 Theoretical Evaluation of nc‐SiO_x_:H as Electron‐ or Hole‐Transport Layer (ETL or HTL)

**Figure 6:**
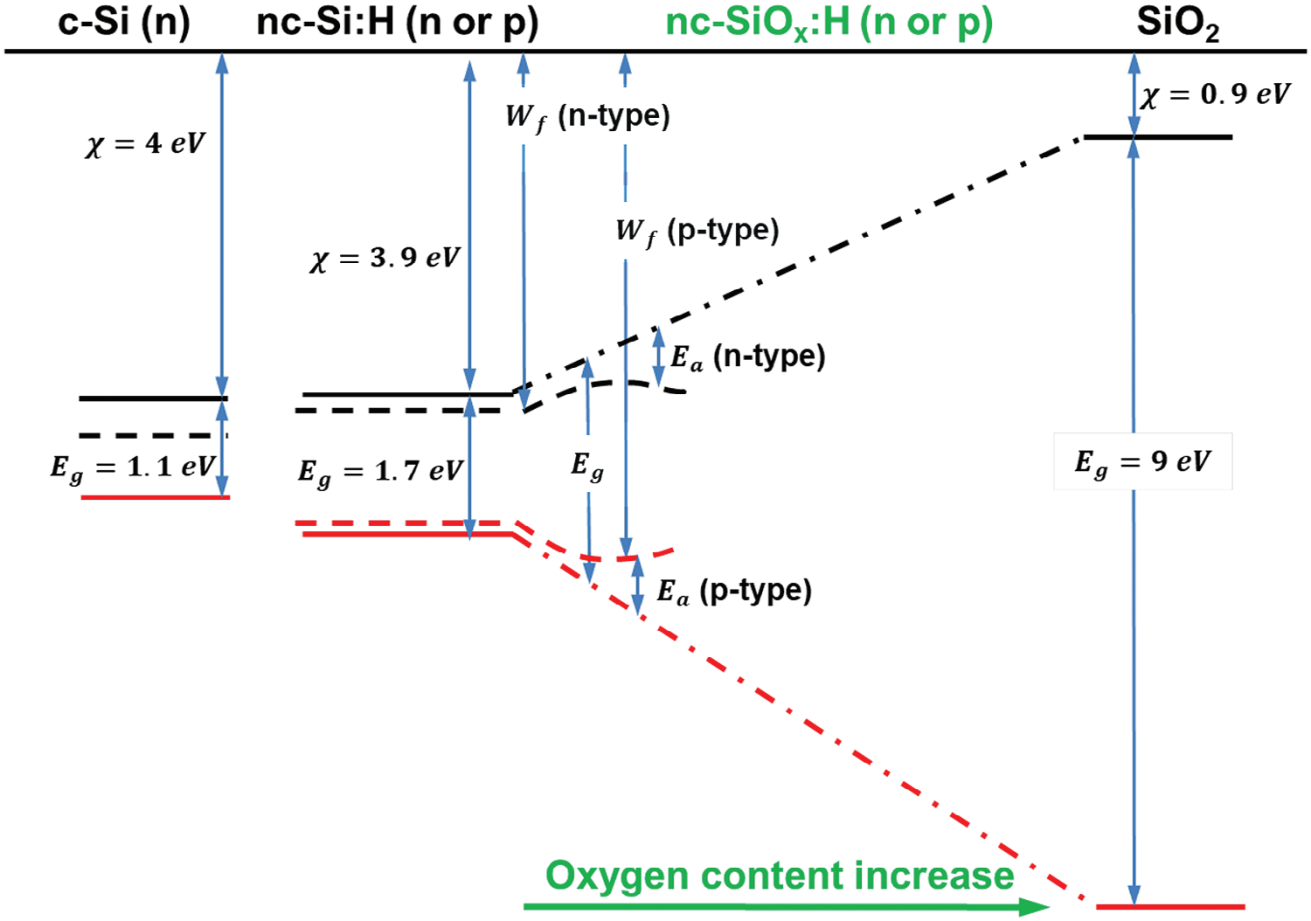
Sketch of energy band of (n‐type) c‐Si bulk isolated from doped nc‐Si:H, doped nc‐SiO_x_:H or SiO_2_. From nc‐Si:H to SiO_2_ we visually vary band gap (E_g_), activation energy (E_a_), and work‐function (W_f_) as a function of oxygen incorporation. Conduction (valence) band energy is illustrated in continuous black (red) line. Changes in the electronic properties of doped nc‐SiO_x_:H layers, such as E_g_, depend on the oxygen content. Note that lateral dimension is not to scale, and E_a_ (dashed lines) illustrates the increasing trend while incorporating oxygen to the layer. Figure adapted from ref. [65]

In terms of the optical and electrical properties, doped nc‐SiO_x_:H materials exhibit tunable band gap (*E*_g_) and activation energy (*E*_a_) depending on the processing conditions. In fact, the former is determined by the oxygen content while the latter is determined by the density of active dopants.^[^ ^23^, ^64^ ^]^ Widening the *E*_g_ entails an change in the energy positioning in the conduction and the valence band of the mixed‐phase material as reported by Biron et al.^[^ ^65^ ^]^ Indeed, the changes in the conduction and valence bands are within 3 eV and 4.3 eV, respectively, as illustrated in **Figure** **6**. Note that the variation of the conduction (valence) band energy is the energy difference between conduction (valence) band energy of doped nc‐Si:H and SiO_2_. Accordingly, increasing the *E*_g_ of doped nc‐SiO_x_:H layers by oxygen incorporation leads to the corresponding increase of the energy band‐offset between c‐Si bulk and doped nc‐SiO_x_:H layers and thus the increase of the potential barriers for carrier collection.^[^ ^66^, ^67^ ^]^ Moreover, the incorporation of oxygen in doped nc‐SiO_x_:H layers inhibits the formation of crystallites and affects the electrical properties due to the increasing *E*_a_ and the decreasing conductivity.^[^ ^65^ ^]^ Interestingly, the combination of wide *E*_g_ (band‐offset) together with low *E*_a_ also affects the work function (*W*_f_). Note that a decrease or increase of *W*_f_ is favorable for n‐ or p‐contacts, respectively.^[^ ^68^, ^69^ ^]^

**Figure 7:**
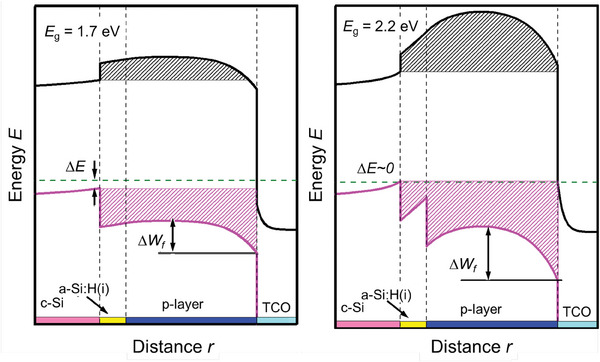
Band diagram at equilibrium of p‐type contact layers featuring the same activation energy (400 meV) but different band gap. Black and pink patterned areas illustrate the energy barriers for electrons and holes, respectively. ΔE and ΔW_f_ indicate the band bending at c‐Si and work‐function mismatch with TCO. p‐type nc‐SiO_x_ layers featuring wider band gap (right) increase the band bending inside c‐Si, but also ΔW_f_. Similar effects are observed for n‐type contact but in the conduction band. Figure is adapted from ref. [67]

**Figure 8:**
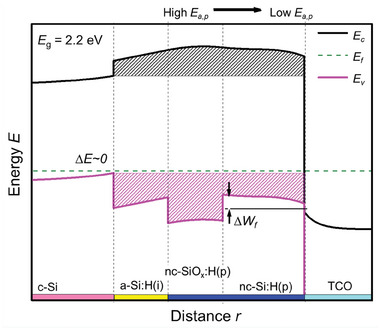
Band diagram at equilibrium of p‐type contact featuring a wide band gap (p‐type nc‐SiO_x_:H, high E_a,p_) incubation layer for p‐type nc‐Si:H (low E_a,p_) to minimize ΔE and reduce ΔW_f_. Black and pink patterned areas illustrate the energy barriers for electrons and holes, respectively. ΔE and ΔW_f_ indicate the band bending at c‐Si and work‐function mismatch with TCO, respectively. Similar effects are observed for n‐contacts but in the conduction band. Figure adapted from ref. [67]

The implications of *W*_f_ variation on the band diagram of c‐Si solar cells are elaborated in **Figure** **7**, depicting a comparison between p‐type contacts based on nanocrystalline materials with different band gap (1.7 eV and 2.2 eV). Wide band gap layers (as doped nc‐SiO_x_:H) are advantageous for carriers selectivity at c‐Si/passivating layer interface because such layers potentially induce a stronger band bending inside c‐Si than plain silicon‐based counterparts.^[^ ^69^, ^70^ ^]^ However, the potential barriers (see patterned areas in Figure 7), could hinder the transport of collecting carriers,^[^ ^67^, ^71^, ^72^, ^73^ ^]^ due to the higher energy band‐offset (high *E*_g_). To reduce such energy barriers for boosting the transport of carriers, the doped layers should be thin enough and/or exhibit low *E*_a_ (i.e., high doping concentration).^[^ ^68^, ^71^, ^74^ ^]^ The proper thickness of doped layers allows an optimal electric field inside the c‐Si and also mitigates any effect from TCO (work‐function).^[^ ^23^, ^64^ ^]^ In fact, depending on the *E*_a_ of the doped layer and the *W*_f_ of the TCO, the thickness of the doped layer can be adjusted for optimal carrier transport as a compromise between band bending inside c‐Si and the potential barrier height.^[^ ^67^ ^]^ Nevertheless, achieving both wide band gap and low activation energy (high active doping level) in thin films based on nc‐SiO_x_:H is technologically not trivia. Experimental work has been aimed at optimizing both layer thickness and doping for efficient carrier collection.^[^ ^8^, ^23^, ^64^, ^75^, ^76^, ^77^, ^78^, ^79^, ^80^ ^]^ However, the interface of doped nc‐SiO_x_:H with TCO is sensitive to inherent properties of nc‐SiO_x_:H, leading to a relatively high built‐in potential and work‐function mismatch (∆*W*_f_). To mitigate such unfavorable effects at the interface with TCO, the addition of a doped silicon layer by deposition or treatment of the interface is effectively used.^[^ ^23^, ^64^ ^]^ **Figure** **8** illustrates the positive effect of using a stack of layers featuring a wide band gap p‐type nc‐SiO_x_:H layer which also acts as incubation layer for the following p‐type nc‐Si:H layer. This approach avoids any possible negative effect from energy misalignment of the doped layer with TCO (see Figure 7, right versus Figure 8).

Similarly, to doped nc‐SiO_x_:H materials, wide *E*_g_ and low *E*_a_ are also pursued in doped polysilicon oxide (poly‐SiO_x_) materials, which are promising candidates to replace highly absorptive polysilicon (poly‐Si) materials in high‐thermal budget carrier‐elective passivating contacts. In fact, absorption in doped poly‐SiO_x_ materials can be lowered with respect to the poly‐Si passivating contacts,^[^ ^81^ ^]^ while heavy doping, with surface doping level above 3 × 10^20^ atm cm^−3^,^[^ ^82^ ^]^ electrically enhances carrier transport and leads to low or negligible *E*_a_. However, due to the incorporation of oxygen, for which amorphous SiO_x_ phase material is in between Si crystals,^[^ ^82^ ^]^ poly‐SiO_x_ is a mixed‐phase material exhibiting lower crystallinity than poly‐Si passivating contacts.^[^ ^44^ ^]^ In particular, the existence of the SiO_x_ phase especially on the surface of the film^[^ ^82^ ^]^ degrades the lateral conductivity of the film and makes it challenging to realize an ohmic contact between the doped poly‐SiO_x_ film and the metallic contact. As a consequence, the use of TCO layers is imperative to build a proper contact enhancing the carrier collection and maintaining the passivation after metallization.^[^ ^44^, ^83^, ^84^ ^]^ In general, similarly to the use of doped nc‐SiO_x_:H films in low‐thermal budget SHJ devices, the advantages of using doped poly‐SiO_x_ layers for carriers’ selectivity and transport in high‐thermal budget SHJ devices depend on the properties of the layers (doping, thickness and ∆*W*_f_ with TCO).

Theoretically, it has been calculated that materials with the electrical properties of nc‐SiO_x_:H layers hold the potential to enable efficiencies >27%^[^ ^67^ ^]^ for single junction solar cells and have already enabled, in the ETL embodiment, a large area (244.5 cm^2^), rear‐junction, low‐thermal budget SHJ solar cell exhibiting power conversion efficiency (*η*) of 26.81%.^[^ ^8^ ^]^ Moreover, the nc‐SiO_x_:H selective contact shows a superior high temperature stability as compared to a‐Si:H selective contacts.^[^ ^85^ ^]^

The use of nc‐SiO_x_:H layers is of particular interest for tandem solar cells applications due to their tunable band gap and refractive index. In fact, such layers can be used as optical interlayers to couple c‐Si bottom cells with a wide variety of top cells, especially those deploying perovskite absorbers. In this type of tandems, nc‐SiO_x_:H materials at the recombination junction between top and bottom cells with appropriate thickness and refractive index regulate the infrared light management demonstrating a current gain in the bottom cell of up to 1.4 mA cm^−2^.^[^ ^21^ ^]^

## 4 Application of nc‐SiO_x_:H on Solar Cell Devices

### 4.1 Silicon Heterojunction Solar Cells

Silicon heterojunction solar cells represent a very promising technology for highly efficient solar cells with relatively low fabrication cost and are predicted to be one of the next mainstream products in the PV industry after passivated emitter and rear cell (PERC).^[^ ^86^, ^87^, ^88^, ^89^, ^90^ ^]^ For conventional SHJ solar cells, intrinsic and doped hydrogenated amorphous silicon layer (a‐Si:H) layer stacks are deposited on both sides of wafers to reduce the surface recombination rate and separate the electron‐hole pairs. TCO layers are deposited on top of the silicon layers to collect charge carriers. The metallization process to apply Ag fingers and busbars is followed to conduct the carriers to the external circuit. However, one of the main conversion efficiency losses for this concept is the photocurrent loss due to parasitic absorption in the front contact layers.^[^ ^91^, ^92^ ^]^ To reduce the parasitic absorption and improve the optical response of SHJ solar cells, the wide band gap material nc‐SiO_x_:H is a suitable candidate to replace the a‐Si:H thin films.^[^ ^93^, ^94^ ^]^ A Considerable amount of research has been done to apply doped nc‐SiO_x_:H layer as carrier selective layers in SHJ solar cells. Some of the reported results are summarized in **Table** **1**.

**Table 1:** Performance of selected SHJ devices featuring nc‐SiO_x_:H or nc‐Si:H (*x* = 0) as carrier selective layers.

Layer stack	*J*_sc_ [mA cm^−2^]	*V*_oc_ [mV]	*FF* [%]	*Η* [%]	Area [cm^2^]	Bif.^a)^	Certi.^b)^	Institute [year]	Ref
nc‐Si:H(p) / nc‐SiO_x_:H(p)	40.40	688	72.9	20.3	1	No	No	HZB [2015]	[95]
nc‐SiO_x_:H(n)	37.04	729	80	21.6	4	No	No	HZB [2017]	[26]
nc‐Si:H(n) / nc‐SiO_x_:H(n) / nc‐Si:H(n)	38.30	731	80.6	22.6	4	No	No	HZB [2018]	[77]
nc‐SiO_x_:H(n); nc‐SiO_x_:H(p)	35.83	682.9	77.9	19.1	0.53	No	Yes	Nankai [2018]	[27]
nc‐SiO_x_:H(n) / nc‐Si:H(n)	39.00	727	77	21.8	10.24	No	No	SKKU [2019]	[96]
nc‐Si:H(n)	39.90	729	79	23.0	4	No	No	HZB [2019]	[97]
nc‐SiO_x_:H(n) / nc‐Si:H(n) ^c)^	38.70	739	80.7	23.1	244.6	Yes	No	FZJ [2019]	[98]
nc‐Si:H(p); nc‐Si:H(n)	39.41	734.1	81.07	23.5	4.029	No	Yes	EPFL [2019]	[99]
nc‐SiO_x_:H(n) ^c)^	39.60	747	84.9	25.1	244.5	Yes	Yes	Hanergy [2019]	[1]
nc‐Si:H(p)	39.48	733	81.4	23.56	4	No	Yes	AIST [2020]	[24]
nc‐Si:H(p) / nc‐SiO_x_:H(p) / nc‐Si:H(p)	39.85	737.5	81.95	24.1	4.06	No	Yes	EPFL [2020]	[2]
nc‐Si:H(p) / nc‐SiO_x_:H(p); nc‐SiO_x_:H(n)	38.85	719.2	80.41	22.5	3.92	No	Yes	TU Delft [2021]	[64]
nc‐SiO_x_:H(n); nc‐SiO_x_:H(p) / a‐SiO_x_:H(i) ^d)^	40.50	729	80	23.6	10.24	Yes	No	SKKU [2021]	[100]
nc‐SiO_x_:H(n)	39.80	731	81.4	23.7	3.61	No	No	FZJ [2021]	[25]
nc‐SiO_x_:H(n) ^c)^	40.24	746	85.08	25.54	274.5	Yes	Yes	Maxwell/ SunDrive [2021]	[101]
nc‐Si:H(p) / nc‐SiO_x_:H(p); nc‐Si:H(n) / nc‐SiO_x_:H(n) / a‐Si:H(n)	39.60	733	81.6	23.7	4	No	No	EPFL [2022]	[102]
nc‐Si:H(n) ^c)^	39.26	741.9	81.96	23.9	244.6	Yes	Yes	FZJ [2022]	[103]
nc‐Si:H(p)	37.9	754	81.5	23.3	4	No	No	AIST [2022]	[3]
nc‐Si:H(n)	39.79	746.8	82.79	24.6	3.9	No	Yes	HZB [2022]	[4]
nc‐Si:H(n) ^c)^	39.40	746	81.7	24.0	3.9	Yes	No	HZB [2022]	[4]
nc‐Si:H(n) / a‐Si:H(n); nc‐SiO_x_:H(p) / nc‐Si:H(p)	39.81	724.5	82.2	23.7	3.92	No	Yes	TU Delft [2022]	[5]
nc‐Si:H(n) / a‐Si:H(n); nc‐SiO_x_:H(p) / nc‐Si:H(p)	39.97	726.0	83.3	24.18	3.92	No	No	TU Delft [2022]	[5]
nc‐Si:H(n) / a‐Si:H(n); nc‐SiO_x_:H(p) / nc‐Si:H(p)^c)^	38.68	719.5	82.07	22.84	3.985	Yes	Yes	TU Delft [2022]	[104]
nc‐SiO_x_:H(n) ^c)^	38.5	745	84.7	24.3	243.36	Yes	No	SIMIT / Zhongwei [2022]	[6]
nc‐SiO_x_:H(n) ^c)^	41.01	750.6	86.08	26.5	274.4	Yes	Yes	LONGi [2022]	[105]
nc‐SiO_x_:H(n); nc‐Si:H(p) ^c)^	40.80	750.2	86.28	26.4	274.5	Yes	Yes	Maxwell/ SunDrive [2022]	[106, 107]
nc‐SiO_x_:H(n) ^c)^	39.98	742.0	85.74	25.44	274.15	Yes	No	IEE/ Huasun [2023]	[108]
nc‐SiO_x_:H(n); nc‐Si:H(p) ^c)^	40.49	747.5	85.71	25.94	274.4	Yes	Yes	Maxwell/Nankai [2023]	[109]
nc‐Si:H(n) / nc‐SiO_x_:H(n); nc‐Si:H(p) ^c)^	41.16	751.1	86.48	26.74	274.4	Yes	Yes	LONGi / SYSU [2023]	[110]
nc‐Si:H(n) / nc‐SiO_x_:H(n); nc‐Si:H(p)	41.45	751.4	86.07	26.81	274.4	No	Yes	LONGi / SYSU [2023]	[110]

^a)^ “Bif.” means “Bifacial”

^b)^ “Certi.” means “Certified”;

c) Front side illumination;

d) Front side one sun illumination, rear side half sun illumination.

**Figure 9:**
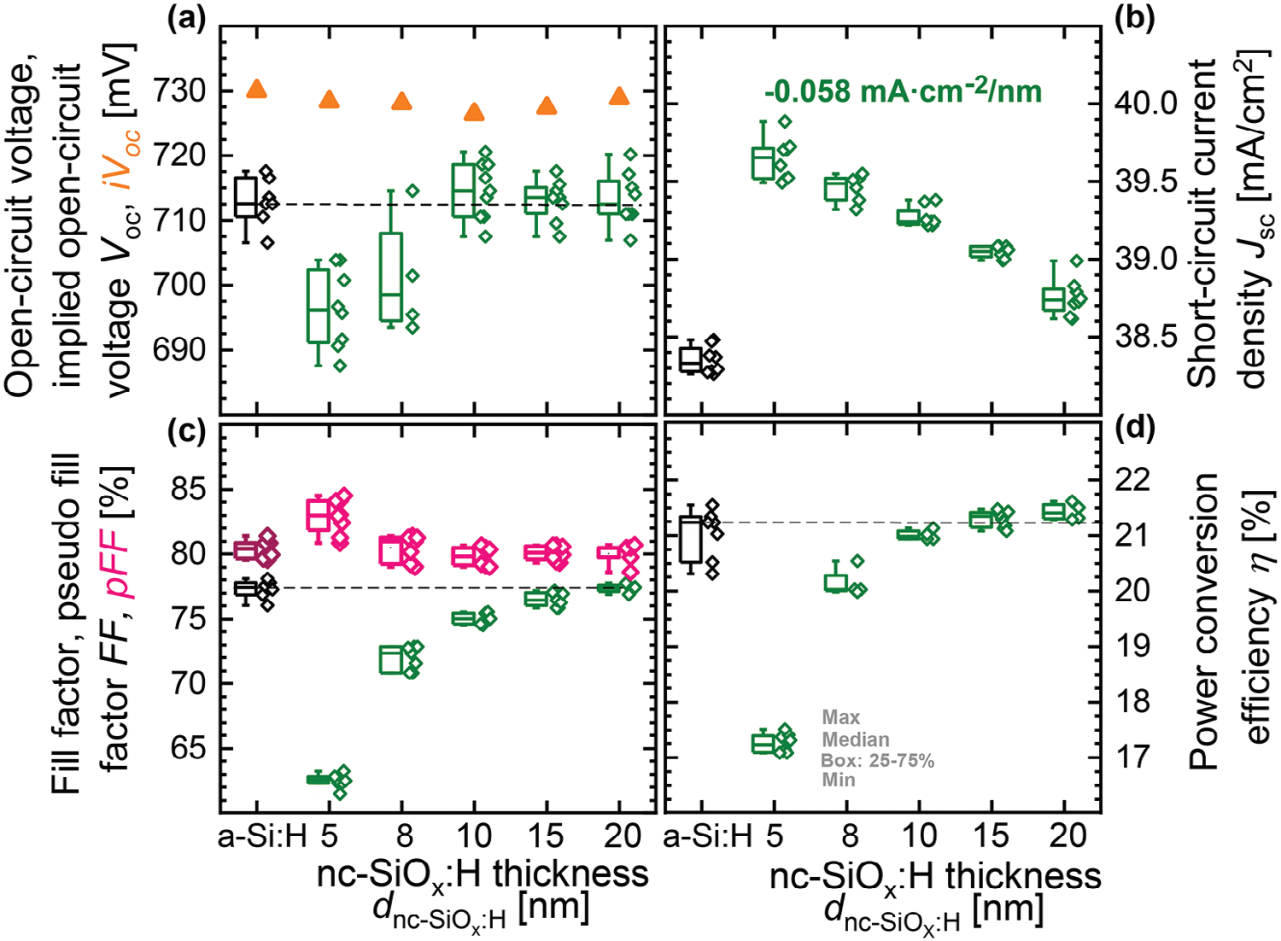
Box plot of a) open‐circuit voltage (*V*_oc_) and implied *V*_oc_ (i*V*_oc_), b) short‐circuit current density (*J*_sc_), c) pseudo fill factor and fill factor (p*FF*, *FF*), and d) power conversion efficiency (*η*) versus the thickness of the nc‐SiO_x_:H layer (d_nc‐SiOx:H_) for cells with nc‐SiO_x_:H (n) single layer.^[^ ^98^ ^]^ A cell with 7 nm a‐Si:H (n) film is used as the reference on the left side.^[^ ^98^ ^]^

With a nc‐SiO_x_:H front contact, a short‐circuit current density (*J*_sc_) above 40 mA cm^−2^ was demonstrated by Mazzarella et al.^[^ ^95^ ^]^ The *J*_sc_ gain in the range of 0.7−1.7 mA cm^−2^ was found when the doped a‐Si:H layers were replaced by doped nc‐SiO_x_:H layers.^[^ ^95^, ^98^, ^111^ ^]^ This increase in *J*_sc_ is due to the improved external quantum efficiency in the short wavelength region. In addition, an improved passivation quality of the silicon layer stacks was achieved when the nc‐SiO_x_:H(n) layer was used instead of the a‐Si:H(n) layer.^[^ ^112^ ^]^ This observation was explained by the more effective field‐effect passivation due to the wider band gap of the nc‐SiO_x_:H layer. **Figure** **9** shows a comparison of the solar cell performance between a‐Si:H and nc‐SiO_x_:H front contact layers. When the nc‐SiO_x_:H thickness (*d*_nc‐SiOx:H_) was decreased from 20 to 10 nm, the open‐circuit voltage (*V*_oc_) of the cells is not affected, but it decreases for nc‐SiO_x_:H (n) layers below 10 nm. The i*V*_oc_ of the cells with nc‐SiO_x_:H (n) layers of different thicknesses are at the same level as the reference cell. Figure 9b shows that the *J*_sc_ increases with decreasing *d*_nc‐SiOx:H_ by −0.058 mA cm^−2^ per nm and the gain of *J*_sc_ is 0.4–1.4 mA cm^−2^ compared to the a‐Si:H reference. Figure 9c shows that the fill factor (*FF*) decreases with reducing *d*_nc‐SiOx:H_ and is more sensitive to the variation of *d*_nc‐SiOx:H_ when it is below 10 nm. In addition, there is an increase in the difference between p*FF* and *FF*, indicating an increase in the series resistance as the *d*_nc‐SiOx:H_ is reduced. Although the power conversion efficiency of the solar cell with 5 nm nc‐SiO_x_:H (n) layer is lower than others (Figure 9d), a high *J*_sc_ of 39.9 mA cm^−2^ and similar i*V*_oc_ as the reference cell can be obtained, indicating a potential to achieve high efficiency with a low series resistance.

As a window layer on the front side of the SHJ solar cell, the layer should be as thin as possible to minimize parasitic absorption as shown in Figure 9b. As a carrier selective layer, the nc‐SiO_x_:H layer should also be thin enough and have a low *E*_a_ to enhance the carrier transport. However, there is a strong correlation between the material properties of nc‐SiO_x_:H and its film thickness. During the growth of nc‐SiO_x_:H thin film, an amorphous incubation layer is grown at the beginning, then the layer is nucleating resulting in the formation of crystallites. Therefore, a reduced crystalline volume fraction and doping efficiency of the nc‐SiO_x_:H layer have been observed with decreasing film thickness, resulting in a decrease in conductivity due to the increase in activation energy.^[^ ^64^, ^113^ ^]^ Although a high crystalline volume fraction is beneficial for the solar cell performance,^[^ ^27^ ^]^ it is a challenging to improve the crystalline volume fraction for a very thin nc‐SiO_x_:H thin film, especially when the doped nc‐SiO_x_:H films are deposited on an intrinsic a‐Si:H passivation layer, which is reported to suppress the nucleation but needed for chemical surface passivation.^[^ ^114^ ^]^

**Figure 10:**
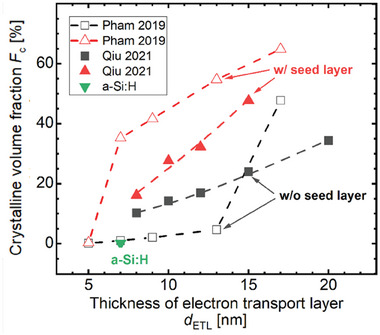
The crystalline volume fraction (*F*_c_) of the doped layers versus the thickness of electron transport layer (*d*_ETL_). The data were collected from literature and the crystalline volume fraction was determined by UV‐Raman spectroscopy^[^ ^98^ ^]^ and Raman spectroscopy.^[^ ^119^ ^]^

In order to promote the crystallization and improve the conductivity of doped nc‐SiO_x_:H thin films, various methods have been reported, such as applying a soft and short CO_2_ plasma treatment^[^ ^31^, ^112^, ^115^ ^]^ which can improve the nucleation due to the oxidized surface, applying a hydrogen plasma treatment,^[^ ^23^ ^]^ reducing the deposition temperature,^[^ ^99^ ^]^ depositing a hydrogenated amorphous silicon oxide buffer layer,^[^ ^116^ ^]^ replacing silane by disilane for the deposition,^[^ ^117^ ^]^ and using a high excitation frequency.^[^ ^118^ ^]^ A common solution is to use a highly crystalline nc‐Si:H seed layer to improve the nucleation of the nc‐SiO_x_:H layer.^[^ ^59^, ^77^, ^119^, ^120^ ^]^ In **Figure** **10** a comparison of the structural properties of nc‐SiO_x_:H with and without nc‐Si:H seed layers is shown. The crystalline volume fraction is significantly improved by the addition of a seed layer, which contributes to highly conductive nc‐SiO_x_:H layers and low activation energies. There are also reports that a doped seed layer performs better than an intrinsic seed layer.^[^ ^121^ ^]^ With the help of nc‐Si:H seed layers, the nc‐SiO_x_:H thin film thickness can be reduced to below 10 nm for the application in SHJ solar cells.^[^ ^77^, ^98^ ^]^

Besides the development of doped nc‐SiO_x_:H for carrier‐selective layers, there are also efforts to replace intrinsic a‐Si:H passivation layer by a‐SiO_x_:H thin films.^[^ ^78^, ^122^, ^123^, ^124^ ^]^ Lifetimes of several milliseconds have been reported using intrinsic a‐SiO_x_:H as the passivation layers for silicon wafers.^[^ ^125^, ^126^ ^]^ Like nc‐SiO_x_:H selective contact layers, a‐SiO_x_:H passivation layers also have the advantage of thermal stability.^[^ ^123^, ^127^ ^]^ The challenge of intrinsic a‐SiO_x_:H passivation lies in the carrier transport. When the passivation layer changes from a‐Si:H to a‐SiO_x_:H, the valence band offset increases from ≈0.3 eV to over 4 eV, indicating that the intrinsic a‐SiO_x_:H layer provides a prominent barrier at the hole contact in SHJ solar cells.^[^ ^128^, ^129^ ^]^ It was demonstrated by Seif et al. that the fill factor of SHJ solar cells decreases rapidly as the thickness of the a‐SiO_x_:H thin film at the hole contact increases.^[^ ^130^ ^]^ On the other hand, an intrinsic a‐SiO_x_:H passivation layer for electron contacts is seldom reported.^[^ ^131^, ^132^, ^133^ ^]^ As more and more SHJ solar cells use the rear junction design and the electron contact becomes the front contact, it gains increasing importance to explore the carrier transport of a‐SiO_x_:H passivation layers for the electron contacts.^[^ ^134^ ^]^

### 4.2 Passivating Contact Solar Cells

Recently, a structure consisting of an ultra‐thin silicon oxide (SiO_x_) stacked with a heavily doped poly‐Si layer has received more and more attention in research institutes and the photovoltaic industry.^[^ ^135^, ^136^, ^137^, ^138^, ^139^, ^140^, ^141^, ^142^, ^143^, ^144^, ^145^ ^]^ This structure is commonly referred to as tunneling oxide passivated contact (TOPCon),^[^ ^135^, ^144^ ^]^ polysilicon on oxide (POLO),^[^ ^138^, ^145^ ^]^ or simply as a poly‐Si passivated contact.^[^ ^139^, ^141^ ^]^ Thanks to the impressive full‐size passivation and excellent carrier‐selective property provided by poly‐Si/SiO_x_ stacks, the champion efficiency of this concept has been increased to 26.1% for both n‐ and p‐type c‐Si solar cells, much higher than conventional PERC devices.^[^ ^142^, ^145^ ^]^ Moreover, the poly‐Si passivation contact technology is compatible with the current mainstream product manufacturing line in the photovoltaic industry, which makes this technology an attractive option for the future upgrade of the PERC mass production lines.^[^ ^146^ ^]^

However, the doped poly‐Si suffers from strong parasitic absorption, quantified as 0.4−0.5 mA cm^−2^ per 10 nm of poly‐Si when placed on the front side.^[^ ^147^, ^148^ ^]^ Thus, poly‐Si/SiO_x_ stacks are commonly used on the rear side of solar cells, but this limits the efficiency potential of devices with poly‐Si junctions. Messmer et al. demonstrated that 0.6%–0.7% efficiency gain can be achieved by applying poly‐Si junctions on both the front (localized n^+^ poly‐Si) and back (full area p^+^ poly‐Si) sides.^[^ ^149^ ^]^ In addition, a strong free carrier absorption in the infrared wavelength range has been reported in poly‐Si layers, resulting in a short‐circuit current density losses of 0.3−0.5 mA cm^−2^ for a 140 nm poly‐Si layer used on the rear side of solar cells.^[^ ^147^, ^150^ ^]^ In order to reduce the optical loss, much effort has been put into the development of carrier‐selective layer alternatives with higher transparency.^[^ ^44^, ^81^, ^82^, ^83^, ^84^, ^151^, ^152^, ^153^, ^154^ ^]^ It has been reported that alloying oxygen into the poly‐Si material promotes the formation of a mixed phase structure, poly‐SiO_x_, resulting in an increase in the band gap and a decrease in the absorption coefficient of the silicon layer.^[^ ^155^, ^156^, ^157^, ^158^ ^]^ Furthermore, simulation results suggested an improved carrier selectivity and an enhancement of the lateral carrier transport at the c‐Si/SiO_x_/poly‐SiO_x_ interface when poly‐Si is replaced by a wider bandgap poly‐SiO_x_ layer.^[^ ^44^, ^157^, ^158^ ^]^ Yang et al. reported an excellent passivation quality and carrier selectivity for both n‐type (i*V*_oc,flat_ = 740 mV, contact resistivity *ρ*_c_ = 0.7mΩ cm^−2^) and p‐type (i*V*_oc,flat_ = 709 mV, *ρ*_c_ = 0.5mΩ cm^−2^) poly‐SiO_x_ layers.^[^ ^84^ ^]^ In addition, the incorporation of oxygen into in poly‐Si could assist the release of stress in the silicon matrix and prevent blistering of the layer during the subsequent high‐temperature annealing process used for crystallization.^[^ ^159^, ^160^ ^]^ Taken together, these advantages of poly‐SiO_x_ suggest an excellent alternative to poly‐Si for reducing optical losses and achieve an improved passivating contact.

The approach for fabricating poly‐SiO_x_/SiO_x_ junctions involves at least four steps: (1) growth of a thin SiO_x_ layer on the wafer surface by thermal or wet‐chemical oxidation; (2) deposition of a‐SiO_x_:H or nc‐SiO_x_:H layers by chemical vapor deposition (CVD) or physical vapor deposition (PVD); (3) recrystallization of the silicon thin films and activation of the dopants by high‐temperature annealing (≥750 °C); and (4) hydrogenation via a forming gas annealing (FGA) treatment or via the deposition of hydrogen‐rich thin films. Many researchers have been devoted themselves to the poly‐SiO_x_/SiO_x_ concept and demonstrated that the process parameters play a major role on the optical, electronic, and passivation properties of the resulting poly‐SiO_x_/SiO_x_ passivating contact.^[^ ^44^, ^81^, ^82^, ^83^, ^84^, ^155^, ^161^, ^162^, ^163^ ^]^ Plasma enhanced chemical vapor deposition (PECVD) is commonly used to prepare the silicon thin film. Similar to the preparation of a‐SiO_x_:H or nc‐SiO_x_:H used in the SHJ technology, CO_2_ was added into the silane‐based plasma during the PECVD process in poly‐SiO_x_ passivating contact technology. By modulating the CO_2_ gas flow ratio, *f*_CO2_ = [CO_2_]/([CO_2_] + [SiH_4_]), the microstructure and the optoelectronic properties of poly‐SiO_x_ layers can be adjusted in a wide range.^[^ ^161^, ^162^ ^]^ As in the case of nc‐SiO_x_:H, lower refractive index, higher optical band gap, lower crystallinity, and lower conductivity of poly‐SiO_x_ layers can be obtained by increasing the amount of O incorporation.^[^ ^161^, ^162^ ^]^

**Figure 11:**
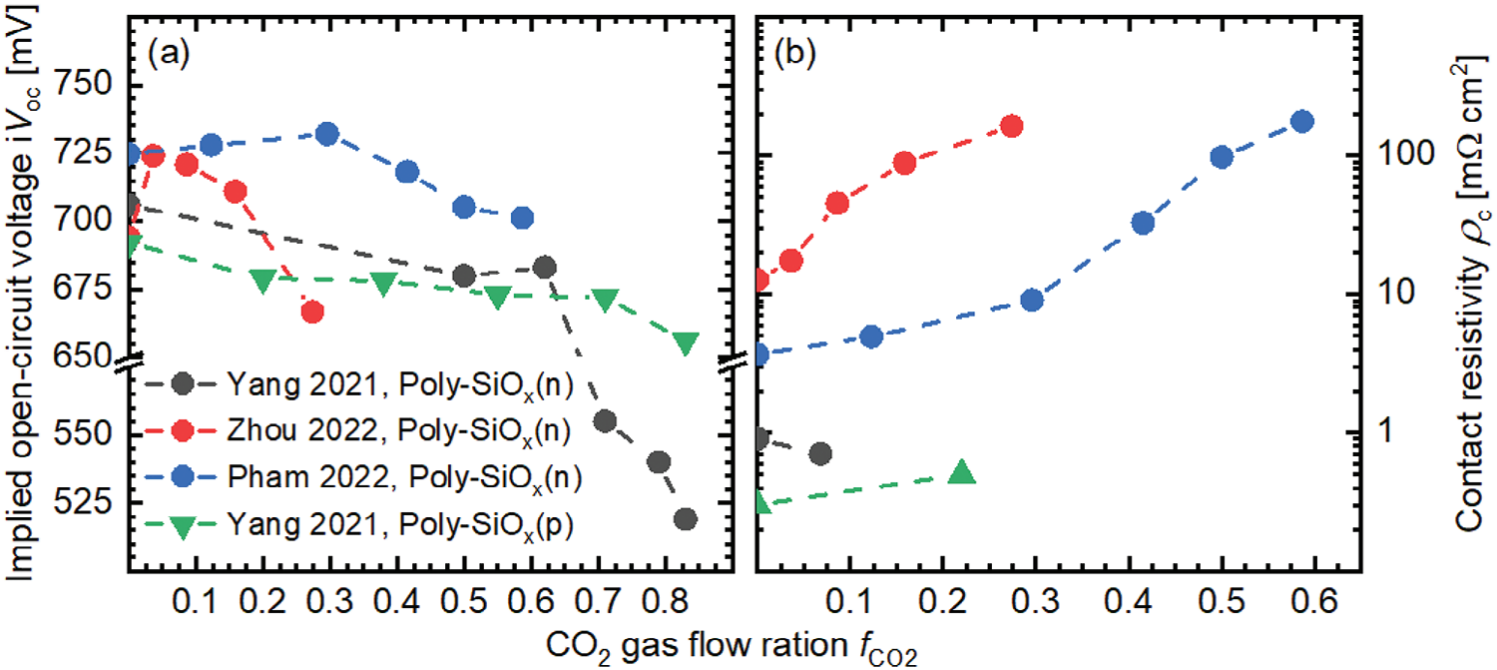
Comparison of a) the implied open‐circuit voltage (i*V*_oc_) and b) the contact resistivity (*ρ*_c_) with respect to the CO_2_ gas flow ratio (*f*_CO2_). The results were selected from literatures.^[^ ^44^, ^84^, ^161^, ^162^ ^]^ Dashed lines serve as the eye guides to eyes.

**Figure** **11** displays the i*V*_oc_ and the contact resistivity (*ρ*_c_) of poly‐SiO_x_/SiO_x_ passivating contacts versus the CO_2_ gas flow ratio. These values were taken from literature.^[^ ^44^, ^84^, ^161^, ^162^ ^]^ Yang et al. observed a degradation of the passivation quality for both n‐ and p‐type poly‐SiO_x_ with increasing the *f*_CO2_.^[^ ^84^ ^]^ However, an increased i*V*_oc_ was reported by Zhou et al. and Pham et al. when introducing a small amount of CO_2_ into the process chamber and then the i*V*_oc_ decreases when further raising *f*_CO2_.^[^ ^161^, ^162^ ^]^ One reason for the improved passivation quality could be the suppressed formation of blisters on the interface when few O is incorporated in the silicon matrix.^[^ ^159^, ^162^ ^]^ Another explanation for the passivation improvement is the additional chemical surface passivation of the widespread a‐SiO_x_ matrix in the mixed phase structure.^[^ ^161^ ^]^ The degradation of i*V*_oc_ with increasing *f*_CO2_ was interpreted by a weaker induced electric field near the c‐Si/SiO_x_ interface due to the reduced doping level in poly‐SiO_x_ layers prepared at higher *f*_CO2_.^[^ ^84^, ^161^, ^162^ ^]^ In terms of contact characteristics, a value of *ρ*_c_ of less than 1mΩ cm^−2^ was observed for n‐ and p‐type poly‐SiO_x_/SiO_x_/c‐Si stacks by Yang et al., as shown in Figure 11b. The contact resistivity increases slightly when *f*_CO2_ falls below a specific value and significantly when further increasing *f*_CO2_ according to the results reported by Zhou et al. and Pham et al.^[^ ^161^, ^162^ ^]^ The worse contact property of poly‐SiO_x_/SiO_x_ is attributed to a deterioration of the silicon crystalline grains or columns, which are the main pathways for the carrier transport in mixed‐phase poly‐SiO_x_. Yang et al. tried to raise the doping gas flow used during PECVD and found an improved passivating contact when adjusting the doping gas flow to a specific value, which could be associated with an enhanced field effect passivation near the wafer surface and more conductive poly‐SiO_x_ layers.^[^ ^84^ ^]^ In addition, Stuckelberger et al. demonstrated that increasing the dopant gas flow rate accumulated the diffusion of dopants from silicon thin film to the wafer and resulted in an increase of the surface dopant concentration by more than one order of magnitude.^[^ ^83^ ^]^

On the other hand, the subsequent high‐temperature annealing treatment is critical to dopant in‐diffusion, contact formation, and interface recombination. It was reported that a declined contact resistivity can be reached by increasing the annealing temperature or prolonging the annealing dwell time, but surface recombination current density first decreased and then increased.^[^ ^82^, ^83^, ^84^ ^]^ It has been demonstrated that larger grains of silicon crystallites in poly‐SiO_x_ layers and a deeper junction can be obtained when raising the annealing temperature from 750 to 950 °C.^[^ ^82^ ^]^ For the surface recombination, there is a tradeoff between the reduction of minority carriers, Auger recombination, and defect creation in the interfacial oxide during the annealing process.^[^ ^83^ ^]^ In addition, the thickness of the poly‐SiO_x_ layer also affects the property of the passivating contact. Several research groups demonstrated that a lower recombination current density (*J*_0_) and contact resistivity can be achieved by thickening the poly‐SiO_x_ layer, however, this is also associated with the loss due to parasitic absorption, especially for devices with poly‐SiO_x_ layer on the illuminated side.^[^ ^82^, ^84^, ^163^ ^]^

**Table** **2** shows the cell performance of devices with poly‐SiO_x_ passivating contact reported in literature. Mack et al. fabricated hybrid cells with a poly‐SiO_x_(n)/SiO_x_ electron‐selective contact on the front side and a silicon heterojunction a‐Si(i/p) hole‐selective contact on the rear, achieving an efficiency of 18.6%.^[^ ^155^ ^]^ The hybrid cell was found to be less temperature sensitive with respect to SHJ cells and the barrier imposed by a SiO_x_/Si‐based contact is less pronounced than the barrier imposed by a standard SHJ contact.^[^ ^155^ ^]^ Pham et al. followed this research and further increased the cell efficiency of this hybrid cell to up to 24%.^[^ ^161^ ^]^ Yang et al. applied n‐ and p‐type doped poly‐SiO_x_ carrier‐selective passivating contacts for both polarities of two‐side contacted solar cells.^[^ ^44^, ^84^ ^]^ The efficiency reached 19.0% with an extremely high FF of 83.5% but a low *J*_sc_ of 33.4 mA cm^−2^ on a flat wafer^[^ ^44^ ^]^ and 20.7% on a textured wafer.^[^ ^84^ ^]^ By placing both carrier‐selective poly‐SiO_x_ junctions on the rear side, an efficiency of 19.7% with 39.3 mA cm^−2^ *J*_sc_ was demonstrated for an interdigitated back contact (IBC) solar cell.^[^ ^44^ ^]^ Zhou et al. replaced n‐type poly‐Si with poly‐SiO_x_ for conventional poly‐Si passivating contact solar cells, achieving a *J*_sc_ of 41.53 mA cm^−2^ and an *η* of 22.6%.^[^ ^162^ ^]^

**Table 2:** Performance of selected solar cells with poly‐SiO_x_ passivating contact.

Layer/stack	*J*_sc_[mA cm^−2^]	*V*_oc_[mV]	*FF* [%]	*Η* [%]	Area [cm^2^]	Bifacial	Certified	Institute [year]	Ref
Poly‐SiO_x_(n); a‐Si:H(p) ^b)^	33.90	691	79.4	18.6	4	No	No	EPFL [2018]	[155]
Poly‐SiO_x_(n); Poly‐SiO_x_(p)	33.40	681	83.5	19.0	2	No	No	TU Delft [2018]	[44]
Poly‐SiO_x_(n); Poly‐SiO_x_(p)^a)^	39.30	650	77	19.7	9	No	No	TU Delft [2018]	[44]
Poly‐SiO_x_(n); Poly‐SiO_x_(p)	39.30	691	76.4	20.7	2	No	No	TU Delft [2021]	[84]
Poly‐SiO_x_(n); Poly‐SiO_x_(p)^b)^	36.68	695	80.3	20.5	3.91	No	Yes	TU Delft [2021]	[33]
Poly‐SiO_x_(n); a‐Si:H(p)^b)^ ^)^	40.90	723	81	24.0	10.24	No	No	SKKU [2021]	[161]
a‐Si:H(p); Poly‐SiO_x_(n)^b)^	38.95	724	75.9	21.4	10.24	Yes	No	SKKU [2022]	[163]
Poly‐SiO_x_(n); P+	41.53	687.8	79.21	22.6	4	No	No	Nankai [2022]	[162]

a) Solar cells prepared with an IBC architecture;

b) Screen printed solar cells.

### 4.3 Perovskite/c‐Si Tandem Solar Cells

Silicon based solar cells are approaching their practical conversion efficiency limit of 29.4%.^[^ ^164^, ^165^ ^]^ To achieve even higher efficiencies, new cell designs are being explored, such as the combination of different band gap solar cells in a tandem device. Wide band gap metal‐halide perovskite solar cells are a perfect match to be used on top of low band gap silicon solar cells in a tandem device. Ideally both cells are processed monolithically on top of each other. The two‐terminal design facilitates low optical and electrical losses as well as lowest production costs.^[^ ^166^, ^167^, ^168^, ^169^, ^170^ ^]^ With such a monolithic tandem based on a wide‐gap perovskite cell deposited on a silicon heterojunction bottom cell, a record efficiency of 29.15% was achieved in 2020,^[^ ^171^ ^]^ and further improved to 29.8% in 2021, which exceeds the efficiency limit of a silicon single junction.^[^ ^172^ ^]^ In 2022 Q. Jeangros et al, from EPFL/CSEM brought the efficiency of perovskite/SHJ tandem cells to 30.93% and 31.3% on a planarized and textured silicon surface, respectively.^[^ ^173^ ^]^ Afterwards, the record efficiency was further improved successively to 32.5% by HZB^[^ ^174^ ^]^ and to 32.7% by KAUST.^[^ ^14^ ^]^ Recently, LONGi announced a new word record efficiency of 34.6% certificated by European Solar Test Installation (ESTI).^[^ ^43^ ^]^

A key challenge in such monolithic tandems remains the integration of the complex layer stack with, for instance, ideal light in‐coupling and low reflection losses. In order to maximize in particular the infrared response of tandem devices several approaches can be used. Ideally a random pyramid textured surface on both sides of the silicon wafer is used, which is industry standard and provides the best light in‐coupling and efficiencies for SHJ solar cells.^[^ ^175^, ^176^, ^177^ ^]^ However, it is still a challenge to conformally deposit a perovskite layer on top of such micrometer sized pyramids especially when using solution‐based processes. In order to reduce the reflection losses at the flat perovskite/silicon interface in the NIR spectral range (Δn (800 nm) >2), a nc‐SiO_x_:H layer was implemented as a medium‐range refractive index interlayer to couple the NIR light into the Si bottom cell, and, thereby, maximize the infrared response.^[^ ^171^, ^178^ ^]^ In this application, the nc‐SiO_x_:H interlayer was tuned, varying the CO_2_ content in the PECVD process to optimize the oxygen content in the layer.^[^ ^19^, ^21^, ^179^ ^]^ As it was mentioned in the previous sections, increasing the oxygen content of a nc‐SiO_x_:H layer decreases the refractive index and conductivity, giving a wide range of options to use this material as optical interlayer between top and bottom cells. A high n > 2.6 and close to zero parasitic absorption in the NIR spectral range combined with excellent electrical contact to both sub‐cells and good lateral conductivity makes this material unique for this application. Its deposition by PECVD at T < 200 °C is compatible with SHJ processing and it can simply replace the regular amorphous silicon n‐layer. To match photocurrent in the tandem devices both the best optical properties of the interlayer and a correctly adjusted perovskite band gap and thickness are needed. As it was analyzed in detail in previous publications, varying the interlayer refractive index by changing its oxygen content demonstrates the ability of properly managing the spectral response in the near infrared region.^[^ ^19^, ^179^ ^]^

**Figure 12:**
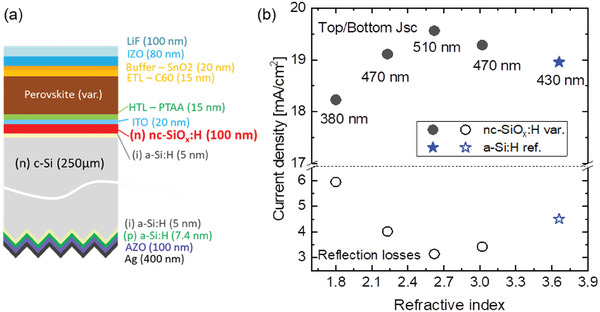
a) Cross‐section structure of the simulated monolithic perovskite/c‐Si tandem solar cell, thicknesses included in the schematics. b) Simulated current density (top) and reflection losses (in mA cm^−^^2^) (bottom) when varying the refractive index (at 800 nm) of the nc‐SiO_x_:H(n) interlayer (100 nm thick) compared to a reference sample without that interlayer using only an a‐Si:H n‐layer (8 nm thick). All data points correspond to a current matched situation obtained by varying the perovskite thickness (values included).^[^ ^180^, ^181^ ^]^

Optical simulations of monolithic perovskite/SHJ tandem solar cells using GenPro4^[^ ^180^ ^]^ demonstrate that the sample using a nc‐SiO_x_:H interlayer with *n* = 2.6 (at 800 nm) provides the maximum current density values. **Figure** **12**a depicts the cross‐section structure of the simulated tandem structure. The optical parameters used in those simulations were experimentally determined for the layers corresponding to the bottom cell including the n layer (i.e., interlayer) and taken from the GenPro4 and PV lighthouse database for the top cell^[^ ^180^, ^181^ ^]^ using a triple cation perovskite material from literature with a band gap >1.6 eV.^[^ ^181^ ^]^ Figure 12b shows the simulated values of current matched perovskite top and SHJ bottom cells when varying the refractive index of the nc‐SiO_x_:H interlayer. Current matching was obtained by varying the perovskite thickness between 380 and 510 nm in the simulation, the corresponding thickness for each nc‐SiO_x_:H interlayer variation is included in the figure. The reflection losses of the samples are also depicted in the lower part of the same graph.

**Figure 13:**
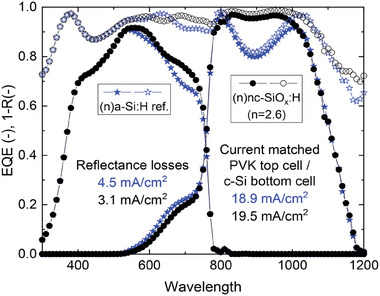
Simulated EQE (filled symbols) and total absorbance (1−R, open symbols) for perovskite/SHJ tandem cells using the best nc‐SiO_x_:H interlayer (black round symbols, 100 nm thick with *n* = 2.6, at 800 nm) and an a‐Si:H reference (blue star symbols, 8 nm).^[^ ^180^, ^181^ ^]^ Both correspond to the current matched situation described in Figure 12. Current density and reflectance losses of both samples are included.

The simulated EQE and the total absorbance curves are plotted in **Figure** **13** for the same samples as in Figure 12: the best nc‐SiO_x_:H sample (*n* = 2.6, black circles) and the a‐Si:H reference sample (blue stars). The optimum refractive index of the nc‐SiO_x_:H interlayer suppresses reflection losses in the spectral region of 800−1050 nm attenuating the minimum in the total absorbance curve (1−R) at ≈850 nm. This leads to a current density gain of 0.6 mA cm^−2^ in the bottom cell with reflection losses reduced by 1.4 mA cm^−2^ compared to the a‐Si:H sample. The advantage of nc‐SiO_x_:H as interlayer in perovskite/Si tandem cells has attracted many researchers all over the world. A lot of R&D efforts on this topic have been done in the past decades and the experimental J‐V parameters of some perovskite/Si tandem cells with nc‐SiO_x_:H layer reported in literature are summarized in **Table** **3**. It is notable that, next to low‐thermal budget SHJ architecture, high‐thermal budget carrier‐selective passivating contacts based on poly‐SiO_x_ were also demonstrated to be a promising architecture for high efficiency perovskite/c‐Si tandem solar cells by Singh et al.^[^ ^37^ ^]^

**Table 3:** Performance of selected two‐terminal perovskite/silicon tandem solar cells featuring nc‐SiO_x_:H or nc‐Si:H (*x*=0) as interconnection layer or carrier selective layer.

Interconnection layer	PVK polarity	Wafer morph.	*J*_sc_ [mA cm^−2^]	*V*_oc_ [mV]	*FF* [%]	*Η* [%]	Area [cm^2^]	Certified	Institute [year]	Ref
ITO / TRL / nc‑SiO_x_:H(p)	p‐i‐n	Flat	15.9	1.71	74.	20.1	0.53	No	Nankai [2018]	[27]
ITO / nc‐SiO_x_:H(n)	p‐i‐n	Flat	18.5	1.76	78.5	25.5	0.77	No	HZB [2018]	[182]
nc‐Si:H(p^+^)/ nc‐Si:H(n^+^)	p‐i‐n	Tex.	19.5	1.79	73.1	25.5	1.42	Yes	EPFL [2018]	[175]
ITO / TRL / nc‑SiO_x_:H(p)	p‐i‐n	Flat	17.1	1.78	74	22.8	0.13	No	Nankai [2018]	[183]
ITO / nc‐SiO_x_:H(p)	p‐i‐n	Flat	16.9	1.75	74	21.9	0.13	No	Tianjin / Nankai [2019]	[184]
ITO / nc‑SiO_x_:H(n)	p‐i‐n	Flat	19.02	1.79	74.6	25.4	1.1	Yes	HZB / Oxford [2019]	[21]
ITO / nc‑SiO_x_:H(n)	p‐i‐n	Flat	19.22	1.76	76.5	26	0.77	No	HZB [2019]	[178]
ITO / nc‑SiO_x_:H(n)	p‐i‐n	Flat	19.23	1.9	79.4	29.2	1.06	Yes	HZB [2020]	[171]
nc‐Si(n)/ITO	p‐i‐n	Tex.	18.46	1.8	75.9	25.2	0.832	Yes	KAUST/ U.Toronto [2021]	[9]
nc‐Si:H(p^+^) / nc‑SiO_x_:H(n)	p‐i‐n	Flat	19.44	1.74	76.73	25.9	1.21	No	Hanergy[2021]	[10]
ITO / nc‑SiO_x_:H(n)	p‐i‐n	Flat	17.81	1.94	80.9	27.9	1	No	HZB [2021]	[185]
ITO / nc‑SiO_x_:H(n)	p‐i‐n	Flat	18.1	1.9	70.1	24.1	4	No	HZB [2022]	[186]
nc‐Si:H(n)	n‐i‐p	Flat	15.71	1.81	75.2	21.43	0.25	No	AIST [2022]	[11]
ITO / nc‑SiO_x_:H(n)	p‐i‐n	Nano‐ textured	19.48	1.92	79.4	29.75	1.0163	Yes	HZB [2022]	[172]
ITO / nc‑SiO_x_:H(n)	p‐i‐n	Tex.	20.11	1.79	79.95	28.84	1.2	Yes	UESTC [2022]	[12]
nc‑Si:H(p)/ nc‑Si:H(n)/ a‐Si:H(n)	p‐i‐n	Tex.	20.473	1.913	79.8	31.25	1.1677	Yes	EPFL/CSEM [2023]	[35]
TCO / nc‑SiO_x_:H(n)	p‐i‐n	Tex.	20.24	1.98	81.18	32.5	1.0139	Yes	HZB[2023]	[174]
IZO / nc‑Si:H(n)	p‐i‐n	Tex.	21.0	1.947	80.0	32.7	1.055	Yes	KAUST[2023]	[14]
nc‑SiO_x_:H(p)/ nc‑SiO_x_:H(n)	p‐i‐n	Tex.	19.82	1.86	73.68	27.16	0.5091	Yes	Nankai [2023]	[15]
ITO/Poly‐SiO_x_(n)	p‐i‐n	Flat	17.8	1.76	74.0	23.18	1	No	TU Delft [2023]	[37]
ITO/ nc‑SiO_x_:H(n)	p‐i‐n	Flat	18.3	1.81	70	23.2	1	No	TU Delft [2023]	[16]

Experimentally, the reduced reflection losses mentioned previously were demonstrated in 2019 in a joint work of HZB with Oxford University, and Oxford PV,^[^ ^21^ ^]^ achieving a tandem conversion efficiency of 25.2% by using SHJ bottom cells from HZB with a 110‐nm thick nc‐SiO_x_:H interlayer having a refractive index of 2.6 (at 800 nm). This cell showed an improvement of 1.4 mA cm^−2^ in short‐circuit current density compared to the reference tandem without nc‐SiO_x_:H interlayer. With an all‐HZB‐made tandem cell by fine tuning the thickness of the perovskite and the TCOs of the perovskite top cell Köhnen et al. improved the current matching and reached a PCE of 26% with a high total current density (top + bottom cell) of 39.5 mA cm^−^^2^ in spite of the flat front surfaces.^[^ ^178^ ^]^ Further optimization of the optical band gap of the perovskite absorber and the hole‐contact layer material later allowed Al‐Ashouri and Köhnen et al. to obtain an almost perfectly current matched tandem cell with an improved open‐circuit voltage yielding an efficiency of 29.15%.^[^ ^171^ ^]^ The formation of the periodic nanotextures on the wafer front side helps Tockhorn and Sutter et al. further improve the short‐circuit current density of the tandem cell, achieving a certified efficiency of 29.8%.^[^ ^172^ ^]^ By combining a triple‐halide perovskite (1.68 electron volt band gap) with a piperazinium iodide interfacial modification, Mariotti and Köhnen et al. improved the band alignment, reduced non‐radiative recombination losses, and enhanced charge extraction at the electron‐selective contact, yielding a certified efficiency of 32.5%.^[^ ^174^ ^]^

## 5 Conclusions

The material properties of nc‐SiO_x_:H and the versatility of its applications in solar cells have been reviewed in this paper. Its favorable optical properties, including a tunable refractive index and wide optical band gaps, have been demonstrated as well as excellent electrical conductivities. We explained the advantages of wide band gap layers (such as doped nc‐SiO_x_:H) for carrier selectivity at c‐Si/passivating layer interface based on a theoretical analysis of charge carrier transport mechanisms in SHJ solar cells. Recent progresses about the fabrication of nc‐SiO_x_:H thin film, such as inserting a nc‐Si:H seed layer for rapid crystalline growth, was discussed. The potential of nc‐SiO_x_:H or a‐SiO_x_:H layers is shown in high temperature applications, such as poly‐SiO_x_ passivating contacts for silicon solar cells. It has been shown that samples using a nc‐SiO_x_:H interlayer with *n* = 2.6 (at 800 nm) provides the highest current density values and could achieve a current density gain of 0.6 mA cm^−2^ in the bottom cell with reflection losses reduced by 1.4 mA cm^−2^ compared to an a‐Si:H sample. By exploiting the unique properties of the nc‐SiO_x_:H material high efficiencies of 26.81% and 32.5% have been achieved for SHJ solar cells and perovskite/c‐Si tandem solar cells, respectively.

## Conflict of Interest

The authors declare no conflict of interest.

## References

[1] X. Ru , M. Qu , J. Wang , T. Ruan , M. Yang , F. Peng , W. Long , K. Zheng , H. Yan , X. Xu , Sol. Energy Mater. Sol. Cells 2020, 215, 110643.

[2] M. Boccard , L. Antognini , V. Paratte , J. Haschke , M. Truong , J. Cattin , J. Dreon , W. Lin , L. L. Senaud , B. Paviet‐Salomon , S. Nicolay , M. Despeisse , C. Ballif , IEEE J. Photovoltaics 2021, 11, 9.

[3] H. Sai , H. Umishio , T. Matsui , Sol. RRL 2021, 5, 2100634.

[4] A. Cruz , D. Erfurt , P. Wagner , A. B. Morales‐Vilches , F. Ruske , R. Schlatmann , B. Stannowski , Sol. Energy Mater. Sol. Cells 2022, 236, 111493.

[5] Y. Zhao , P. Procel , A. Weeber , M. Zeman , O. Isabella , Prog. Photovoltaics 2022, 31, 1

[6] Y. Yang , W. Liu , L. Zhang , S. Huang , X. Li , K. Jiang , Z. Li , Z. Yan , S. Lan , X. Wu , Z. Ma , Y. Zhou , Z. Liu , Mater. Lett. 2022, 309, 131360.

[7] C. Yu , K. Gao , C. W. Peng , C. He , S. Wang , W. Shi , V. Allen , J. Zhang , D. Wang , G. Tian , Y. Zhang , W. Jia , Y. Song , Y. Hu , J. Colwell , C. Xing , Q. Ma , H. Wu , L. Guo , G. Dong , H. Jiang , H. Wu , X. Wang , D. Xu , K. Li , J. Peng , W. Liu , D. Chen , A. Lennon , X. Cao , et al., Nat. Energy 2023, 8, 1375.

[8] H. Lin , M. Yang , X. Ru , G. Wang , S. Yin , F. Peng , C. Hong , M. Qu , J. Lu , L. Fang , C. Han , P. Procel , O. Isabella , P. Gao , Z. Li , X. Xu , Nat. Energy 2023, 8, 789.

[9] M. De Bastiani , A. J. Mirabelli , Y. Hou , F. Gota , E. Aydin , T. G. Allen , J. Troughton , A. S. Subbiah , F. H. Isikgor , J. Liu , L. Xu , B. Chen , E. Van Kerschaver , D. Baran , B. Fraboni , M. F. Salvador , U. W. Paetzold , E. H. Sargent , S. De Wolf , Nat. Energy 2021, 6, 167.

[10] Y. He , Z. Tang , L. Mao , S. Yang , T. Yang , M. Xie , Q. Chang , L. Ding , B. He , C. Peng , C. Yu , X. Hao , J. Zhang , K. Zheng , C. Han , Y. Zhang , H. Yan , X. Xu , Phys. Status Solidi – Rapid Res. Lett. 2021, 15, 2100119.

[11] Y. Kato , H. Katayama , T. Kobayashi , M. Kozawa , Y. Nishigaki , T. Kobayashi , Y. Kinden , K. Oiwake , R. Ishihara , T. Matsui , Y. Aya , T. Hashiguchi , D. Kanematsu , A. Terakawa , H. Fujiwara , Prog. Photovolt. Res. Appl. 2022, 30, 1198.

[12] L. Mao , T. Yang , H. Zhang , J. Shi , Y. Hu , P. Zeng , F. Li , J. Gong , X. Fang , Y. Sun , X. Liu , J. Du , A. Han , L. Zhang , W. Liu , F. Meng , X. Cui , Z. Liu , M. Liu , Adv. Mater. 2022, 34, 2206193. 10.1002/adma.202206193 35985840

[13] S. Mariotti , E. Köhnen , F. Scheler , K. Sveinbjörnsson , L. Zimmermann , M. Piot , F. Yang , B. Li , J. Warby , A. Musiienko , D. Menzel , F. Lang , S. Kessler , I. Levine , D. Mantione , A. Al‐Ashouri , M. S. Härtel , K. Xu , A. Cruz , J. Kurpiers , P. Wagner , H. Köbler , J. Li , A. Magomedov , D. Mecerreyes , E. Unger , A. Abate , M. Stolterfoht , B. Stannowski , R. Schlatmann , et al., Science 2023, 381, 63. 37410849 10.1126/science.adf5872

[14] E. Aydin , E. Ugur , B. K. Yildirim , T. G. Allen , P. Dally , A. Razzaq , F. Cao , L. Xu , B. Vishal , A. Yazmaciyan , A. A. Said , S. Zhumagali , R. Azmi , M. Babics , A. Fell , C. Xiao , S. De Wolf , Nature 2023, 623, 732. 37769785 10.1038/s41586-023-06667-4

[15] Y. Li , X. Wang , Q. Xu , Y. Li , Y. Zhang , W. Han , C. Sun , Z. Zhu , Q. Huang , B. Shi , Y. Zhao , X. Zhang , Sol. Energy Mater. Sol. Cells 2023, 262, 112539.

[16] Y. Zhao , K. Datta , N. Phung , A. E. A. Bracesco , V. Zardetto , G. Paggiaro , H. Liu , M. Fardousi , R. Santbergen , P. P. Moya , C. Han , G. Yang , J. Wang , D. Zhang , B. T. Van Gorkom , T. A. Van Der Pol , M. Verhage , M. M. Wienk , W. M. M. Kessels , A. Weeber , M. Zeman , L. Mazzarella , M. Creatore , R. A. J. Janssen , O. Isabella , ACS Appl. Energy Mater. 2023, 6, 5217. 37234970 10.1021/acsaem.3c00136 PMC10206623

[17] A. Lambertz , V. Smirnov , T. Merdzhanova , K. Ding , S. Haas , G. Jost , R. E. I. Schropp , F. Finger , U. Rau , Sol. Energy Mater. Sol. Cells 2013, 119, 134.

[18] A. Richter , V. Smirnov , A. Lambertz , K. Nomoto , K. Welter , K. Ding , Sol. Energy Mater. Sol. Cells 2018, 174, 196.

[19] L. Mazzarella , A. Morales‐Vilches , L. Korte , R. Schlatmann , B. Stannowski , Coatings 2020, 10, 1.

[20] H. Tan , P. Babal , M. Zeman , A. H. M. Smets , Sol. Energy Mater. Sol. Cells 2015, 132, 597.

[21] L. Mazzarella , Y.‐H. Lin , S. Kirner , A. B. Morales‐Vilches , L. Korte , S. Albrecht , E. Crossland , B. Stannowski , C. Case , H. J. Snaith , R. Schlatmann , Adv. Energy Mater. 2019, 9, 1803241.

[22] D. Qiu , W. Duan , A. Lambertz , K. Bittkau , U. Rau , K. Ding , Sol. RRL 2024, 8, 2400095.

[23] Y. Zhao , L. Mazzarella , P. Procel , C. Han , G. Yang , A. Weeber , M. Zeman , O. Isabella , Prog. Photovoltaics Res. Appl. 2020, 28, 425.

[24] H. Umishio , H. Sai , T. Koida , T. Matsui , Prog. Photovoltaics Res. Appl. 2021, 29, 344.

[25] D. Qiu , W. Duan , A. Lambertz , Z. Wu , K. Bittkau , K. Qiu , Z. Yao , U. Rau , K. Ding , ACS Appl. Energy Mater. 2021, 4, 7544.

[26] L. Mazzarella , A. B. Morales‐Vilches , M. Hendrichs , S. Kirner , L. Korte , R. Schlatmann , B. Stannowski , IEEE J. Photovoltaics 2018, 8, 70.

[27] Q. Ren , S. Li , S. Zhu , H. Ren , X. Yao , C. Wei , B. Yan , Y. Zhao , X. Zhang , Sol. Energy Mater. Sol. Cells 2018, 185, 124.

[28] A. Lambertz , T. Grundler , F. Finger , J. Appl. Phys. 2011, 109, 113109.

[29] J. Sritharathikhun , F. Jiang , S. Miyajima , A. Yamada , M. Konagai , Jpn. J. Appl. Phys. 2009, 48, 1016031.

[30] K. Ding , T. Kirchartz , B. E. Pieters , C. Ulbrich , A. M. Ermes , S. Schicho , A. Lambertz , R. Carius , U. Rau , Sol. Energy Mater. Sol. Cells 2011, 95, 3318.

[31] L. Mazzarella , S. Kirner , O. Gabriel , L. Korte , B. Stannowski , B. Rech , R. Schlatmann , Energy Procedia 2015, 77, 304.

[32] W. Liu , Y. Liu , Z. Yang , C. Xu , X. Li , S. Huang , J. Shi , J. Du , A. Han , Y. Yang , G. Xu , J. Yu , J. Ling , J. Peng , L. Yu , B. Ding , Y. Gao , K. Jiang , Z. Li , Y. Yang , Z. Li , S. Lan , H. Fu , B. Fan , Y. Fu , W. He , F. Li , X. Song , Y. Zhou , Q. Shi , et al., Nature 2023, 617, 717. 37225883 10.1038/s41586-023-05921-z PMC10208971

[33] I. Hwang , Y. Jeong , Y. Shiratori , J. Park , S. Miyajima , I. Yoon , K. Seo , Cell Reports Phys. Sci. 2020, 1, 100242.

[34] J. Yoon , A. J. Baca , S.‐I. Park , Elvikis , J. B. Geddes , L. Li , R. H. Kim , J. Xiao , S. Wang , T.‐H. Kim , M. J. Motala , B. Y. Ahn , E. B. Duoss , J. A. Lewis , R. G. Nuzzo , P. M. Ferreira , Y. Huang , A. Rockett , J. A. Rogers , Nat. Mater. 2008, 7, 907. 18836435 10.1038/nmat2287

[35] X. Y. Chin , D. Turkay , J. A. Steele , S. Tabean , S. Eswara , M. Mensi , P. Fiala , C. M. Wolff , A. Paracchino , K. Artuk , D. Jacobs , Q. Guesnay , F. Sahli , G. Andreatta , M. Boccard , Q. Jeangros , C. Ballif , Science 2023, 381, 59. 37410835 10.1126/science.adg0091

[36] S. De Wolf , E. Aydin , Science 2023, 381, 30. 37410846 10.1126/science.adi6278

[37] M. Singh , K. Datta , A. Amarnath , F. Wagner , Y. Zhao , G. Yang , A. Bracesco , N. Phung , D. Zhang , V. Zardetto , M. Najafi , S. C. Veenstra , G. Coletti , L. Mazzarella , M. Creatore , M. M. Wienk , R. A. J. Janssen , A. W. Weeber , M. Zeman , O. Isabella , Prog. Photovoltaics Res. Appl. 2023, 31, 877.

[38] P. Tockhorn , J. Sutter , A. Cruz , P. Wagner , K. Jäger , D. Yoo , F. Lang , M. Grischek , B. Li , J. Li , O. Shargaieva , E. Unger , A. Al‐Ashouri , E. Köhnen , M. Stolterfoht , D. Neher , R. Schlatmann , B. Rech , B. Stannowski , S. Albrecht , C. Becker , Nat. Nanotechnol. 2022, 17, 1214. 36280763 10.1038/s41565-022-01228-8 PMC9646483

[39] P. Wagner , P. Tockhorn , L. Zimmermann , E. Köhnen , S. Mariotti , F. Scheler , M. Härtel , S. Albrecht , L. Korte , Sol. RRL 2024, 8, 2300963.

[40] P. Wagner , P. Tockhorn , S. Hall , S. Albrecht , L. Korte , Sol. RRL 2023, 7, 2200954.

[41] At 26.81%, LONGi sets a new world record efficiency for silicon solar cells, https://www.longi.com/en/news/propelling‐the‐transformation/ (accessed: November 2022).

[42] LONGi Sets New World‐Record for Silicon Solar Cell Efficiency, Launching 2nd Generation Ultra‐Efficient BC‐Based Module, https://www.longi.com/en/news/longi‐hi‐mo9‐bc‐world‐record/ (accessed: May 2024).

[43] V. Shaw , Longi claims 34.6% efficiency for perovskite‐silicon tandem solar cell, PV Magazine, 2024.

[44] G. Yang , Guo , P. Procel , A. Weeber , O. Isabella , M. Zeman , Appl. Phys. Lett. 2018, 112, 193904.

[45] T. Matsushita , T. Aoki , T. Otsu , H. Yamoto , H. Hayashi , M. Okayama , Y. Kawana , Jpn. J. Appl. Phys. 1976, 15, 35

[46] P. Sichanugrist , T. Yoshida , Y. Ichikawa , H. Sakai , J. Non. Cryst. Solids 1993, 164‐166, 1081.

[47] A. Lambertz , Development of Doped Microcrystalline Silicon Oxide and its Application to Thin‐Film Silicon Solar Cells, University Utrecht 2015.

[48] T. Kilper , W. Beyer , G. Bräuer , T. Bronger , R. Carius , M. N. Van Den Donker , D. Hrunski , A. Lambertz , T. Merdzhanova , A. Mück , B. Rech , W. Reetz , R. Schmitz , U. Zastrow , A. Gordijn , J. Appl. Phys. 2009, 105, 74509.

[49] K. Y. Chan , D. Knipp , A. Gordijn , H. Stiebig , J. Appl. Phys. 2008, 104, 1.

[50] L. Houben , Plasmaabscheidung von mikrokristallinem Silizium: Merkmale und Mikrostruktur und deren Deutung im Sinne von Wachstumsvorgängen, Universität Düsseldorf 1999.

[51] O. Vetterl , F. Finger , R. Carius , P. Hapke , L. Houben , O. Kluth , A. Lambertz , A. Mück , B. Rech , H. Wagner , Sol. Energy Mater. Sol. Cells 2000, 62, 97

[52] L. Houben , M. Luysberg , Hapke , R. Carius , F. Finger , H. Wagner , Philos. Mag. A Phys. Condens. Matter, Struct. Defects Mech. Pro 1998, 77, 1447.

[53] V. Smirnov , A. Lambertz , S. Moll , M. Bär , D. E. Starr , R. G. Wilks , M. Gorgoi , A. Heidt , M. Luysberg , B. Holländer , F. Finger , Phys. Status Solidi Appl. Mater. Sci. 2016, 213, 1814.

[54] M. Luysberg , P. Hapke , R. Carius , F. Finger , Philos. Mag. A Phys. Condens. Matter, Struct. Defects Mech. Pro 1997, 75, 31.

[55] T. Itoh , K. Yamamoto , K. Ushikoshi , S. Nonomura , S. Nitta , J. Non. Cryst. Solids 2000, 266–269, 201.

[56] M. Della Noce , E. Bobeico , L. Lancellotti , L. V. Mercaldo , I. Usatii , D. Veneri , AIP Conf. Proc. 2019, 2147, 040003.

[57] D. Qiu , Development of industry‐scalable processes for nanocrystalline silicon oxide in silicon heterojunction solar cells, RWTH Aachen 2023.

[58] S. Samanta , D. Das , Curr. Appl. Phys. 2021, 23, 42.

[59] S. Kim , S. M. Iftiquar , C. Shin , J. Park , J. Yi , Mater. Chem. Phys. 2019, 229, 392.

[60] M. Klingsporn , S. Kirner , C. Villringer , D. Abou‐Ras , I. Costina , M. Lehmann , B. Stannowski , J. Appl. Phys. 2016, 119, 223104.

[61] A. Richter , L. Zhao , F. Finger , K. Ding , IEEE 42nd Photovolt. Spec. Conf. PVSC, IEEE, Piscataway, NJ 2015, 2.

[62] A. Lambertz , F. Finger , B. Holländer , J. K. Rath , R. E. I. Schropp , J. Non. Cryst. Solids 2012, 358, 1962.

[63] R. Janssen , A. Janotta , D. Dimova‐Malinovska , M. Stutzmann , Phys. Rev. B – Condens. Matter Mater. Phys. 1999, 60, 13561.

[64] Y. Zhao , P. Procel , C. Han , L. Mazzarella , G. Yang , A. Weeber , M. Zeman , O. Isabella , Sol. Energy Mater. Sol. Cells 2021, 219, 110779.

[65] R. Biron , C. Pahud , F. J. Haug , J. Escarré , K. Söderström , C. Ballif , J. Appl. Phys. 2011, 110, 124511.

[66] R. Woods‐Robinson , A. N. Fioretti , J. Haschke , M. Boccard , K. A. Persson , C. Ballif , IEEE J. Photovoltaics 2021, 11, 247.

[67] P. Procel , G. Yang , O. Isabella , M. Zeman , Sol. Energy Mater. Sol. Cells 2018, 186, 66.

[68] S. M. Sze , K. K. Ng , Physics of Semiconductor Devices, Wiley, New York 2006

[69] M. Bivour , J. Temmler , H. Steinkemper , M. Hermle , Sol. Energy Mater. Sol. Cells 2015, 142, 34.

[70] M. Bivour , C. Reichel , M. Hermle , S. W. Glunz , Sol. Energy Mater. Sol. Cells 2012, 106, 11.

[71] M. K. Ieong , M. Solomon , S. E. Laux , H. S. Wong , D. Chidambarrao , Tech. Dig.–Int. Electron Devices Meet, IEEE, Piscataway, NJ 1998, 733.

[72] P. Procel , G. Yang , O. Isabella , M. Zeman , IEEE J. Photovoltaics 2019, 9, 374.

[73] H. Steinkemper , F. Feldmann , M. Bivour , M. Hermle , Energy Procedia 2015, 77, 195.

[74] A. Gehring , S. Selberherr , IEEE Trans. Device Mater. Reliab. 2004, 4, 306.

[75] D.‐W. Kang , Sichanugrist , H. Zhang , M. Konagai , Prog. Photovoltaics Res. Appl. 2017, 25, 384.

[76] K. Ding , U. Aeberhard , V. Smirnov , B. Holländer , F. Finger , U. Rau , Jpn. J. Appl. Phys. 2013, 52, 122304.

[77] L. Mazzarella , A. B. Morales‐Vilches , L. Korte , R. Schlatmann , B. Stannowski , Sol. Energy Mater. Sol. Cells 2018, 179, 386.

[78] K. Ding , U. Aeberhard , F. Finger , U. Rau , J. Appl. Phys. 2013, 113, 134501.

[79] K. Nakada , S. Miyajima , M. Konagai , Jpn. J. Appl. Phys. 2015, 54, 082301.

[80] H. Fujioka , T. Krajangsang , Sichanugrist , M. Konagai , Conf. Rec. IEEE Photovolt. Spec. Conf. 2011, 3, 2570.

[81] M. Singh , R. Santbergen , L. Mazzarella , A. Madrampazakis , G. Yang , R. Vismara , Z. Remes , A. Weeber , M. Zeman , O. Isabella , Sol. Energy Mater. Sol. Cells 2020, 210, 1.

[82] J. Stuckelberger , G. Nogay , Wyss , Q. Jeangros , C. Allebé , F. Debrot , X. Niquille , M. Ledinsky , A. Fejfar , M. Despeisse , F. J. Haug , Löper , C. Ballif , Sol. Energy Mater. Sol. Cells 2016, 158, 2. 10.1021/acsami.6b12714 27959489

[83] J. Stuckelberger , Loper , C. Ballif , G. Nogay , Wyss , A. Ingenito , C. Allebe , J. Horzel , B. A. Kamino , M. Despeisse , F. J. Haug , IEEE J. Photovoltaics 2018, 8, 389.

[84] G. Yang , C. Han , P. Procel , Y. Zhao , M. Singh , L. Mazzarella , M. Zeman , O. Isabella , Prog. Photovoltaics Res. Appl. 2021, 30, 141.

[85] H. A. Gatz , J. K. Rath , M. A. Verheijen , W. M. M. Kessels , R. E. I. Schropp , Phys. Status Solidi Appl. Mater. Sci. 2016, 213, 1932.

[86] G. M. Wilson , M. Al‐Jassim , W. K. Metzger , S. W. Glunz , Verlinden , G. Xiong , L. M. Mansfield , B. J. Stanbery , K. Zhu , Y. Yan , J. J. Berry , A. J. Ptak , F. Dimroth , B. M. Kayes , A. C. Tamboli , R. Peibst , K. Catchpole , M. O. Reese , C. S. Klinga , Denholm , M. Morjaria , M. G. Deceglie , J. M. Freeman , M. A. Mikofski , D. C. Jordan , G. Tamizhmani , D. B. Sulas‐Kern , J. Phys. D. Appl. Phys. 2020, 53, 493001.

[87] M. Hermle , F. Feldmann , M. Bivour , J. C. Goldschmidt , S. W. Glunz , Appl. Phys. Rev. 2020, 7, 021305.

[88] M. Fischer , M. Woodhouse , S. Herritsch , J. Trude , International technology roadmap for photovoltaic (ITRPV), https://itrpv.vdma.org/en/ueber‐uns (accessed: April 2020).

[89] A. Louwen , W. Van Sark , R. Schropp , A. Faaij , Sol. Energy Mater. Sol. Cells 2016, 147, 295.

[90] W. Duan , A. Lambertz , K. Bittkau , D. Qiu , K. Qiu , U. Rau , K. Ding , Prog. Photovoltaics Res. Appl. 2021, 30, 384.

[91] W. Duan , K. Bittkau , A. Lambertz , K. Qiu , Z. Yao , P. Steuter , D. Qiu , U. Rau , K. Ding , Sol. RRL 2021, 5, 2000576.

[92] Z. C. Holman , A. Descoeudres , L. Barraud , F. Z. Fernandez , J. Seif , S. De Wolf , C. Ballif , IEEE J. Photovoltaics 2012, 2, 7.

[93] H. A. Gatz , Y. Kuang , M. A. Verheijen , J. K. Rath , W. M. M. (E). Kessels , R. E. I. Schropp , MRS Proc 2015, 1770, 7.

[94] C. Lei , C. W. Peng , J. Zhong , H. Li , M. Yang , K. Zheng , X. Qu , L. Wu , C. Yu , Y. Li , X. Xu , Sol. Energy Mater. Sol. Cells 2020, 209, 110439.

[95] L. Mazzarella , S. Kirner , B. Stannowski , L. Korte , B. Rech , R. Schlatmann , Appl. Phys. Lett. 2015, 106, 23902.

[96] S. Kim , J. Park , D. Phong , C. Shin , S. M. Iftiquar , J. Yi , Sci. Rep 2018, 8, 1. 30006606 10.1038/s41598-018-28823-x PMC6045650

[97] A. B. Morales‐Vilches , A. Cruz , S. Pingel , S. Neubert , L. Mazzarella , D. Meza , L. Korte , R. Schlatmann , B. Stannowski , IEEE J. Photovoltaics 2019, 9, 34.

[98] D. Qiu , W. Duan , A. Lambertz , K. Bittkau , P. Steuter , Y. Liu , A. Gad , M. Pomaska , U. Rau , K. Ding , Sol. Energy Mater. Sol. Cells 2020, 209, 110471.

[99] A. N. Fioretti , M. Boccard , R. Monnard , C. Ballif , IEEE J. Photovoltaics 2019, 9, 1158.

[100] D. Pham , S. Lee , Y. Kim , J. Yi , J. Phys. Chem. Solids 2021, 154, 110059.

[101] T. Tang , C. Yu , C.‐W. Peng , G. Dong , C. He , X. Ran , H. Jiang , V. Allen , X. Cao , J. Zhou , Prog. Photovoltaics Res. Appl. 2022, 31, 449.

[102] L. Antognini , C. Sthioul , J. Dréon , V. Paratte , D. Türkay , L. L. Senaud , C. Ballif , M. Boccard , Sol. Energy Mater. Sol. Cells 2022, 248, 111975.

[103] D. Qiu , W. Duan , A. Lambertz , A. Eberst , K. Bittkau , U. Rau , K. Ding , Sol. RRL 2022, 6, 1.

[104] C. Han , R. Santbergen , M. Van Duffelen , P. Procel , Y. Zhao , G. Yang , X. Zhang , M. Zeman , L. Mazzarella , O. Isabella , Prog. Photovoltaics Res. Appl. 2022, 30, 750.

[105] LONGi once again sets new world record for HJT solar, https://www.longi.com/en/news/new‐hjt‐world‐record/ (accessed: June 2022).

[106] 26.41%! The Ultra‐high‐efficiency Microcrystalline HJT Cells have been Jointly Developed by Maxwell and SunDrive, https://www.maxwell‐gp.com/en/news‐center/maxwell/434.html (accessed: September 2022).

[107] C. Yu , K. Gao , C. W. Peng , C. He , S. Wang , W. Shi , V. Allen , J. Zhang , D. Wang , G. Tian , Y. Zhang , W. Jia , Y. Song , Y. Hu , J. Colwell , C. Xing , Q. Ma , H. Wu , L. Guo , G. Dong , H. Jiang , H. Wu , X. Wang , D. Xu , K. Li , J. Peng , W. Liu , D. Chen , A. Lennon , X. Cao , et al., Nat. Energy 2023, 8, 1375.

[108] L. Wen , L. Zhao , G. Wang , X. Jia , X. Xu , S. Qu , X. Li , X. Zhang , K. Xin , J. Xiao , W. Wang , Sol. Energy Mater. Sol. Cells 2023, 258, 112429.

[109] C. Yu , Q. Zou , Q. Wang , Y. Zhao , X. Ran , G. Dong , C. W. Peng , V. Allen , X. Cao , J. Zhou , Y. Zhao , X. Zhang , Nat. Energy 2023, 8, 1119.

[110] H. Lin , M. Yang , X. Ru , G. Wang , S. Yin , F. Peng , C. Hong , M. Qu , J. Lu , L. Fang , C. Han , P. Procel , O. Isabella , P. Gao , Z. Li , X. Xu , Nat. Energy 2023, 8, 789.

[111] Y. Zhang , C. Yu , M. Yang , H. Yan , J. Zhang , X. Xu , IEEE 42nd Photovolt. Spec. Conf. PVSC, IEEE, Piscataway, NJ 2015.

[112] L. Mazzarella , S. Kirner , O. Gabriel , S. S. Schmidt , L. Korte , B. Stannowski , B. Rech , R. Schlatmann , Phys. Status Solidi Appl. Mater. Sci. 2017, 214, 1532958.

[113] Y. Zhang , R. Cong , W. Zhao , Y. Li , C. Jin , W. Yu , G. Fu , Sci. Bull. 2016, 61, 787.

[114] P. Roca I Cabarrocas , N. Layadi , T. Heitz , B. Drévillon , I. Solomon , Appl. Phys. Lett. 1995, 66, 3609. 10.1103/physrevb.52.5136 9981697

[115] M. Boccarda , R. Monnard , L. Antognini , C. Ballif , AIP Conf. Proc. 2018, 1999, 40003.

[116] G. Bugnon , G. Parascandolo , S. Hänni , M. Stuckelberger , M. Charrière , M. Despeisse , F. Meillaud , C. Ballif , Sol. Energy Mater. Sol. Cells 2014, 120, 143.

[117] J. Seif , A. Descoeudres , G. Nogay , S. Hanni , S. M. De Nicolas , N. Holm , J. Geissbuhler , A. Hessler‐Wyser , M. Duchamp , R. E. Dunin‐Borkowski , M. Ledinsky , S. De Wolf , C. Ballif , IEEE J. Photovoltaics 2016, 6, 1132.

[118] Y. Djeridane , A. Abramov , P. Roca I Cabarrocas , Thin Solid Films 2007, 515, 7451.

[119] D. Pham , S. Kim , S. Lee , A. H. T. Le , E. C. Cho , J. Park , J. Yi , Infrared Phys. Technol. 2019, 102, 103037.

[120] D. Pham , S. Kim , S. Kim , S. Lee , A. H. T. Le , J. Park , J. Yi , Mater. Sci. Semicond. Process. 2019, 96, 1.

[121] C. W. Peng , C. Lei , T. Ruan , J. Zhong , M. Yang , W. Long , C. Yu , Y. Li , X. Xu , IEEE 46th Photovolt. Spec. Conf., IEEE, Chicago, Illinois, 2019, 2550.

[122] T. Krajangsang , S. Inthisang , J. Sritharathikhun , A. Hongsingthong , A. Limmanee , S. Kittisontirak , Chinnavornrungsee , R. Phatthanakun , K. Sriprapha , Thin Solid Films 2017, 628, 107.

[123] L. Serenelli , L. Martini , F. Menchini , M. Izzi , E. Bobeico , I. Usatii , R. Asquini , D. Caputo , G. de Cesare , P. D. Veneri , M. Tucci , IEEE 7th World Conf. on Photovolt. Energy Conversion (WCPEC) (A Joint Conf. of 45th IEEE PVSC, 28th PVSEC & 34th EU PVSEC), IEEE, Piscataway, NJ 2018, 3108.

[124] J. F. Chen , S. S. Zhao , L. L. Yan , H. Z. Ren , C. Han , D.‐K. Zhang , C. C. Wei , G. C. Wang , G.‐F. Hou , Y. Zhao , X. D. Zhang , Chinese Phys. B 2020, 29, 38801.

[125] M. Inaba , S. Todoroki , K. Nakada , S. Miyajima , Jpn. J. Appl. Phys. 2016, 55, 04ES04.

[126] L. Martini , L. Serenelli , F. Menchini , M. Izzi , R. Asquini , G. de Cesare , D. Caputo , M. Tucci , Proceeding of 33rd Eur. Photovolt. Sol. Energy Conf. Exhib., Amsterdam, the Netherlands 2017, 773.

[127] L. Mazzarella , E. S. Arinze , B. Qiu , N. Palmquist , Y. Cheng , Y. Lin , G. Nyirjesy , G. Qian , S. M. Thon , IEEE 44th Photovolt. Spec. Conf. PVSC, IEEE, Piscataway, NJ 2017, 667.

[128] M. Liebhaber , M. Mews , T. F. Schulze , L. Korte , B. Rech , K. Lips , Appl. Phys. Lett. 2015, 106, 31601.

[129] M. Mews , M. Liebhaber , B. Rech , L. Korte , Appl. Phys. Lett. 2015, 107, 13902.

[130] J. Peter Seif , A. Descoeudres , M. Filipič , F. Smole , M. Topič , Z. Charles Holman , S. De Wolf , C. Ballif , J. Appl. Phys. 2014, 115, 24502.

[131] D. Deligiannis , J. Van Vliet , R. Vasudevan , R. A. C. M. M. Van Swaaij , M. Zeman , J. Appl. Phys. 2017, 121.

[132] B. Zhang , Y. Zhang , R. Cong , Y. Li , W. Yu , G. Fu , Sol. Energy 2017, 155, 670.

[133] H. Zhang , K. Nakada , M. Konagai , Thin Solid Films 2017, 628, 214.

[134] M. Bivour , S. Schröer , M. Hermle , S. W. Glunz , Sol. Energy Mater. Sol. Cells 2014, 122, 120.

[135] F. Feldmann , M. Bivour , C. Reichel , M. Hermle , S. W. Glunz , Proceeding of 28th Eur. Photovolt. Sol. Energy Conf. Exhib., Paris, France, 2013, 988.

[136] D. Yan , A. Cuevas , J. I. Michel , C. Zhang , Y. Wan , X. Zhang , J. Bullock , Joule 2021, 5, 811.

[137] Z. Yao , G. Yang , C. Han , Moya , E. Özkol , J. Yan , Y. Zhao , L. Cao , R. Van Swaaij , L. Mazzarella , O. Isabella , Sol. RRL 2023, 7, 2300186.

[138] U. Römer , R. Peibst , T. Ohrdes , B. Lim , J. Krügener , E. Bugiel , T. Wietler , R. Brendel , Sol. Energy Mater. Sol. Cells 2014, 131, 85.

[139] S. Li , M. Pomaska , J. Hoß , J. Lossen , F. Pennartz , M. Nuys , R. Hong , A. Schmalen , J. Wolff , F. Finger , U. Rau , K. Ding , Appl. Phys. Lett. 2019, 114, 153901.

[140] S. Li , M. Pomaska , J. Hoß , J. Lossen , M. Ziegner , R. Hong , F. Finger , U. Rau , K. Ding , ACS Appl. Mater. Interfaces 2019, 11, 30493. 31361110 10.1021/acsami.9b10360

[141] W. Chen , J. Stuckelberger , W. Wang , S. Phang , D. Macdonald , Y. Wan , D. Yan , Sol. Energy Mater. Sol. Cells 2021, 232, 111356.

[142] Cell Sets Our New Record with Maximum Conversion Efficiency of 26.1%, https://www.jinkosolar.com/en/site/newsdetail/1775 (accessed: October 2022).

[143] J. B. Heng , J. Fu , B. Kong , Y. Chae , W. Wang , Z. Xie , A. Reddy , K. Lam , C. Beitel , C. Liao , C. Erben , Z. Huang , Z. Xu , IEEE J. Photovoltaics 2015, 5, 82.

[144] F. Feldmann , J. Schön , J. Niess , W. Lerch , M. Hermle , Sol. Energy Mater. Sol. Cells 2019, 200, 109978.

[145] F. Haase , C. Hollemann , S. Schäfer , A. Merkle , M. Rienäcker , J. Krügener , R. Brendel , R. Peibst , Sol. Energy Mater. Sol. Cells 2018, 186, 184.

[146] S. W. Glunz , B. Steinhauser , J.‐I. Polzin , C. Luderer , B. Grübel , T. Niewelt , A. M. O. M. Okasha , M. Bories , H. Nagel , K. Krieg , F. Feldmann , A. Richter , M. Bivour , M. Hermle , Prog Photovolt Res Appl 2021, 31, 341.

[147] S. Reiter , N. Koper , R. Reineke‐Koch , Y. Larionova , M. Turcu , J. Krügener , D. Tetzlaff , T. Wietler , U. Höhne , J. D. Kähler , R. Brendel , R. Peibst , Energy Procedia 2016, 92, 199.

[148] F. Feldmann , C. Reichel , R. Müller , M. Hermle , Sol. Energy Mater. Sol. Cells 2017, 159, 265.

[149] C. Messmer , A. Fell , F. Feldmann , N. Wöhrle , J. Schön , M. Hermle , IEEE Journal 2020, 10, 335.

[150] F. Feldmann , M. Nicolai , R. Müller , C. Reichel , M. Hermle , Energy Procedia 2017, 124, 31.

[151] J. Benick , A. Richter , R. Müller , H. Hauser , F. Feldmann , P. Krenckel , S. Riepe , F. Schindler , M. C. Schubert , M. Hermle , A. W. Bett , S. W. Glanz , IEEE J. Photovoltaics 2017, 7, 1171.

[152] G. Nogay , C. Ballif , A. Ingenito , E. Rucavado , Q. Jeangros , J. Stuckelberger , P. Wyss , M. Morales‐Masis , F. J. Haug , Loper , IEEE J. Photovoltaics 2018, 8, 1478.

[153] M. Köhler , M. Pomaska , P. Procel , R. Santbergen , A. Zamchiy , B. Macco , A. Lambertz , W. Duan , Cao , B. Klingebiel , S. Li , A. Eberst , M. Luysberg , K. Qiu , O. Isabella , F. Finger , T. Kirchartz , U. Rau , K. Ding , Nat. Energy 2021, 6, 529.

[154] A. Eberst , A. Zamchiy , K. Qiu , Winkel , H. T. Gebrewold , A. Lambertz , W. Duan , S. Li , K. Bittkau , T. Kirchartz , U. Rau , K. Ding , Sol. RRL 2022, 6, 1.

[155] I. Mack , J. Stuckelberger , P. Wyss , G. Nogay , Q. Jeangros , J. Horzel , C. Allebé , M. Despeisse , F. J. Haug , A. Ingenito , P. Löper , C. Ballif , Sol. Energy Mater. Sol. Cells 2018, 181, 9.

[156] D. Das , S. Samanta , Mater. Chem. Phys. 2020, 243, 122628.

[157] O. Isabella , G. Yang , P. Procel , M. Zeman , patent 2017E00057 NL, 2017.

[158] R. Santbergen , G. Yang , P. Procel , G. Limodio , A. Weeber , O. Isabella , M. Zeman , Light, Energy and the Environment, Optica Publishing Group, Washington, DC 2017, paper PW3A.5.

[159] S. Choi , O. Kwon , K. H. Min , M. S. Jeong , K. T. Jeong , M. G. Kang , S. Park , K. K. Hong , H. E. Song , K.‐H. Kim , Sci. Rep. 2020, 10, 9672. 32541851 10.1038/s41598-020-66801-4 PMC7295771

[160] S. Dutta , S. Chatterjee , K. Mallem , Y. H. Cho , J. Yi , Renew. Energy 2019, 144, 2.

[161] D. Pham , D. Oh , V.‐A. Dao , Y. Kim , J. Yi , Appl. Mater. Today 2022, 29, 101604.

[162] J. Zhou , X. Su , Q. Huang , Y. Zeng , D. Ma , W. Liu , B. Yan , J. Ye , J. Yang , X. Zhang , H. Jin , Y. Zhao , G. Hou , Nano Energy 2022, 98, 107319.

[163] M. Q. Khokhar , S. Q. Hussain , S. Chowdhury , M. A. Zahid , D. Pham , S. Jeong , S. Kim , S. Kim , E. C. Cho , J. Yi , Energy Convers. Manag. 2022, 252, 115033.

[164] W. Shockley , H. J. Queisser , J. Appl. Phys. 1961, 32, 510.

[165] A. Richter , M. Hermle , S. W. Glunz , IEEE J. Photovoltaics 2013, 3, 1184.

[166] Z. Zhu , K. Mao , J. Xu , J. Energy Chem. 2021, 58, 219.

[167] E. Raza , Z. Ahmad , Energy Reports 2022, 8, 5820.

[168] M. Jošt , L. Kegelmann , L. Korte , S. Albrecht , Adv. Energy Mater. 2020, 10, 1904102.

[169] F. Fu , J. Li , T. C.‐J. Yang , H. Liang , A. Faes , Q. Jeangros , C. Ballif , Y. Hou , Adv. Mater. 2022, 34, 2106540. 10.1002/adma.202106540 35060205

[170] A. W. Y. Ho‐Baillie , J. Zheng , M. A. Mahmud , F.‐J Ma , D. R. Mckenzie , M. A. Green , Appl. Phys. Rev. 2021, 8, 041307.

[171] A. Al‐Ashouri , E. Köhnen , B. Li , A. Magomedov , H. Hempel , P. Caprioglio , J. A. Márquez , A. Belen Morales Vilches , E. Kasparavicius , J. A. Smith , N. Phung , D. Menzel , M. Grischek , L. Kegelmann , D. Skroblin , C. Gollwitzer , T. Malinauskas , M. Jošt , G. Matič , B. Rech , R. Schlatmann , M. Topič , L. Korte , A. Abate , B. Stannowski , D. Neher , M. Stolterfoht , T. Unold , V. Getautis , S. Albrecht , Science. 2020, 370, 1300. 33303611 10.1126/science.abd4016

[172] P. Tockhorn , J. Sutter , A. Cruz , P. Wagner , K. Jäger , D. Yoo , F. Lang , M. Grischek , B. Li , J. Li , O. Shargaieva , E. Unger , A. Al‐Ashouri , E. Köhnen , M. Stolterfoht , D. Neher , R. Schlatmann , B. Rech , B. Stannowski , S. Albrecht , C. Becker , Nat. Nanotechnol. 2022, 17, 1214. 36280763 10.1038/s41565-022-01228-8 PMC9646483

[173] New world records: Perovskite‐on‐silicon‐tandem solar cells, https://www.csem.ch/en/press/new‐world‐reco.rds‐perovskite‐on‐silicon‐tandem‐solar (accessed: July 2022).

[174] S. Mariotti , E. Köhnen , F. Scheler , K. Sveinbjörnsson , L. Zimmermann , M. Piot , F. Yang , B. Li , J. Warby , A. Musiienko , D. Menzel , F. Lang , S. Keßler , I. Levine , D. Mantione , A. Al‐Ashouri , M. S. Härtel , K. Xu , A. Cruz , J. Kurpiers , Wagner , H. Köbler , J. Li , A. Magomedov , D. Mecerreyes , E. Unger , A. Abate , M. Stolterfoht , B. Stannowski , R. Schlatmann , et al., Suppl. Inf. 2023, 381, 63. 10.1126/science.adf5872 37410849

[175] F. Sahli , J. Werner , B. A. Kamino , M. Bräuninger , R. Monnard , B. Paviet‐Salomon , L. Barraud , L. Ding , J. J. Diaz Leon , D. Sacchetto , G. Cattaneo , M. Despeisse , M. Boccard , S. Nicolay , Q. Jeangros , B. Niesen , C. Ballif , Nat. Mater. 2018, 17, 820. 29891887 10.1038/s41563-018-0115-4

[176] B. Chen , Z. J. Yu , S. Manzoor , S. Wang , W. Weigand , Z. Yu , G. Yang , Z. Ni , X. Dai , Z. C. Holman , J. Huang , Joule 2020, 4, 850.

[177] J. Chen , A. D. Brunner , J. Z. Cogan , J. K. Nuñez , A Fields , B. Adamson , D. N. Itzhak , J. Y. Li , M. Mann , M. D. Leonetti , J. S. Weissman , Science 2020, 367, 1140. 32139545 10.1126/science.aay0262 PMC7289059

[178] E. Köhnen , M. Jošt , A. B. Morales‐Vilches , P. Tockhorn , A. Al‐Ashouri , B. Macco , L. Kegelmann , L. Korte , B. Rech , R. Schlatmann , B. Stannowski , S. Albrecht , Sustain. Energy Fuels 2019, 3, 1995.

[179] L. Mazzarella , M. Werth , K. Jäger , M. Jošt , L. Korte , S. Albrecht , R. Schlatmann , B. Stannowski , Opt. Express 2018, 26, A487. 29801255 10.1364/OE.26.00A487

[180] R. Santbergen , T. Meguro , T. Suezaki , G. Koizumi , K. Yamamoto , M. Zeman , IEEE J. Photovoltaics 2017, 7, 919.

[181] E. Raoult , R. Bodeux , S. Jutteau , S. Rives , Eur. Photovolt. Sol. Energy Conf. Exhib. (EU PVSEC) 2019, 36, 757.

[182] M. Jošt , E. Köhnen , A. B. Morales‐Vilches , B. Lipovšek , K. Jäger , B. Macco , A. Al‐Ashouri , J. Krč , L. Korte , B. Rech , R. Schlatmann , M. Topič , B. Stannowski , S. Albrecht , Energy Environ. Sci. 2018, 11, 3511.

[183] S. Zhu , F. Hou , W. Huang , X. Yao , B. Shi , Q. Ren , J. Chen , L. Yan , S. An , Z. Zhou , H. Ren , C. Wei , Q. Huang , Y. Li , G. Hou , X. Chen , Y. Ding , G. Wang , B. Li , Y. Zhao , X. Zhang , RRL Solar 2018, 2, 1.

[184] F. Hou , C. Han , O. Isabella , L. Yan , B. Shi , J. Chen , S. An , Z. Zhou , W. Huang , H. Ren , Q. Huang , G. Hou , X. Chen , Y. Li , Y. Ding , G. Wang , C. Wei , D. Zhang , M. Zeman , Y. Zhao , X. Zhang , Nano Energy 2019, 56, 234.

[185] E. Köhnen , P. Wagner , F. Lang , A. Cruz , B. Li , M. Roß , M. Jošt , A. B. Morales‐Vilches , M. Topič , M. Stolterfoht , D. Neher , L. Korte , B. Rech , R. Schlatmann , B. Stannowski , S. Albrecht , Sol. RRL 2021, 5, 1.

[186] Z. Peng , K. Xu , A. C. Bournazou , E. Unger , S. Albrecht , B. Stannowski , AIP Conf. Proc. 2023, 2826, 90003.

